# Soft, Reactive, and Alive: A Dynamic Framework for Degradation and Functional Stability of Polymeric Biomaterials

**DOI:** 10.3390/polym18172108

**Published:** 2026-08-30

**Authors:** Alfredo Rondinella, Elia Marin

**Affiliations:** 1 Department Polytechnic of Engineering and Architecture, University of Udine, 33100 Udine, Italy; 2 Biomaterials Engineering Laboratory, Kyoto Institute of Technology, Sakyo-ku, Matsugasaki, Kyoto 606-8585, Japan; 3 Biomedical Research Center, Kyoto Institute of Technology, Sakyo-ku, Matsugasaki, Kyoto 606-8585, Japan

**Keywords:** polymeric biomaterials, degradation mechanisms, dynamic biocompatibility, functionality retention

## Abstract

Polymeric biomaterials do not degrade in vivo through isolated chemical or mechanical events. Hydrolysis, oxidation, enzymatic cleavage, fatigue, wear, protein adsorption, and lipid uptake often interact across the material surface, bulk, and surrounding biological environment, producing time-dependent changes in both structure and function. Here, we propose a conceptual framework for organizing polymer degradation under biomedical conditions as a directional network of coupled mechanisms, emphasizing how established degradation pathways can influence one another and collectively contribute to time-dependent functional loss. The framework distinguishes degradation reactions from interfacial modulators and links molecular damage to functionality retention, defined according to application-specific endpoints such as mechanical strength, mass retention, dimensional stability, or interfacial performance. We introduce a qualitative interaction matrix to describe how individual mechanisms can amplify or modulate downstream pathways, and we use this matrix to identify recurrent degradation archetypes across major biomedical polymer classes, including polyesters, polyolefins, polyamides, polyurethanes, silicones, polyacrylates, polyvinyl polymers, polyimides, and natural polymers. This perspective emphasizes that material optimization strategies rarely eliminate degradation; instead, they shift the hierarchy of active pathways. By reframing biocompatibility as a dynamic, functionality-dependent property, the proposed framework provides a structured basis for comparing polymeric biomaterials, designing more realistic in vitro tests, and developing future data-driven models of long-term implant performance.

## 1. Introduction

The concept of biocompatibility is, by nature, relative and dynamic rather than absolute. Contrary to the conventional view of a stable equilibrium between a material and the human body, no material, particularly no polymeric biomaterial, ever achieves a true thermodynamic equilibrium once implanted in a biological environment [1,2,3,4]. Implantation initiates a sequence of time-dependent processes such as molecular adsorption, cellular recruitment and tissue remodeling that continuously redefine the material–host relationship [5,6,7]. From the very first seconds after contact with blood or interstitial fluids, the material surface becomes a rapidly evolving bio-interface formed by adsorption and conformational changes in proteins, lipids and ions, which governs subsequent biological events [7,8,9]. The organism “sees” a moving target rather than a fixed material identity. Therefore, biocompatibility cannot be reduced to a single material attribute, but should be framed as the outcome of an evolving, non-equilibrium system in which both the host response and the material itself adapt over multiple time scales, from seconds to years, and where “compatibility” depends on context (implant site, patient condition, loading, and intended function) [3,5,10].

Polymeric biomaterials exemplify this dynamic behavior: they exhibit a broad range of morphologies, from predominantly amorphous to semicrystalline structures, with their permeability, chain mobility, and susceptibility to chemical and mechanical degradation strongly influenced by crystallinity, molecular architecture, and processing history [11,12,13]. Polymeric biomaterials may undergo water uptake and swelling, plasticization, physical aging, stress relaxation, and microstructural rearrangements. Simultaneously, they can experience chemical evolution such as hydrolysis, oxidation, chain scission, leaching of residual monomers/additives, enzymatically mediated degradation and, in load-bearing applications, surface wear [12,13,14,15,16]. These transformations alter local pH, osmolarity, and mechanical cues, feeding back into cell behavior and extracellular matrix deposition [5,17]. Such processes rarely occur in isolation. They are interdependent and self-amplifying. Protein adsorption can catalyze hydrolysis, oxidation can embrittle the surface and promote wear, while mechanical abrasion can expose new, chemically active interfaces [5,17,18,19,20,21].

It is important to distinguish that biocompatibility is primarily a surface-driven phenomenon, governed by the very first molecular interactions with the biological milieu [5,22,23,24], whereas the bulk structure of the material dictates its mechanical lifetime and structural integrity [24,25]. In practice, “surface” should be understood not only as the outermost chemistry (top 1–10 nm) [26,27], but as a dynamically evolving interphase that may extend to micrometric depths through swelling, diffusion of water/ions, and oxidative penetration [28,29,30,31]. Crucially, the bulk and the surface of a material are not independent. Bulk-driven deformation and damage can alter topography and expose new functional groups, while surface-driven chemical aging can propagate inward and compromise bulk mechanical performance [32,33]. Wear is a surface phenomenon with bulk consequences, representing a crucial bridge between these two domains and is capable of both generating bioactive debris and continuously reshaping the surface chemistry exposed to the biological environment [20,31,32,34,35]. Through abrasion, adhesion, and fatigue wear mechanisms, the material repeatedly “renews” its interface, stripping away conditioning films and exposing fresh polymer chains, fillers, or oxidized layers to the environment [36,37,38]. At the same time, wear particles (whose size, shape, and chemistry depend on the underlying bulk microstructure and properties) can act as potent biological signals, being phagocytosed and triggering inflammasome activation and cytokine release, thereby amplifying periprosthetic inflammation and osteolysis in load-bearing settings [39,40,41].

Understanding the behavior of polymers therefore requires acknowledging the complexity and variability of the biological environment itself. Body fluids are not uniform media. Their molecular composition, pH, ionic strength, protein concentration, and oxidative potential vary substantially depending on anatomical location, tissue type, physiological condition, and even local inflammation [42,43,44,45,46,47]. Consequently, each implant, in each patient, exists within a unique and temporally evolving microenvironment. This has two important implications for polymeric biomaterials. First, in vitro testing captures only a subset of relevant parameters [48,49]. Second, “biocompatibility” and durability should be interpreted probabilistically rather than deterministically, as distributions of responses shaped by patient-to-patient variability [50,51]. Even within the same individual, acute post-implantation phases (protein adsorption and inflammation), remodeling and long-term steady states (encapsulation, chronic low-grade inflammation, or integration) present distinct chemical and mechanical boundary conditions that can shift the dominant degradation mechanism over time [5,7,50,52,53,54].

However, this variability should not discourage attempts to model or generalize. Several simplifications are both possible and meaningful. The physiological temperature range is narrow (≈36–38 °C) [55,56], allowing thermal degradation to be safely neglected for most polymers [57,58]. Likewise, because the vast majority of polymeric implants are internal devices, photo-oxidation can be excluded as a relevant degradation pathway [59,60]. Under these conditions, hydrolysis, oxidation, and enzymatic degradation remain the dominant processes dictating the chemical evolution of the material in vivo via Arrhenius-type behavior, although each mechanism can be specific for polymer classes and environments [29,57,61].

In addition, the mechanical properties of polymers are another important aspect in deciding the suitability of these materials for medical applications, beyond the chemical behavior [62,63,64]. Compared with metals and ceramics, polymers possess unique mechanical advantages. Their low modulus of elasticity enables them to match the tissues in terms of mechanical properties, thus reducing stress shielding and improving load transfer [65,66]. Their high deformability and resilience, on the other hand, make them ideal candidates for flexible devices, scaffolds, and articulating surfaces where elasticity and energy absorption are desirable characteristics [65,67,68]. At the same time, the mechanical properties of polymers can be easily tailored to different applications through chemistry and structure (molecular weight, crystallinity, cross-linking density, copolymerization ratio, and reinforcing agents), leading designers to modify stiffness, extensibility, and toughness to match the specific application [63,68,69,70,71].

However, these advantages come at the cost of reduced mechanical reliability. Polymers exhibit lower fatigue strength, creep resistance, and wear durability than metallic or ceramic counterparts [72,73,74]. Their performance is strongly time- and rate-dependent. Under constant load, they may creep; under cyclic loading, they may accumulate damage; and under sustained deformation, they may undergo stress relaxation behaviors that are often negligible in other classes of materials but central for polymers [74,75]. Repeated mechanical loading can lead to microcrack propagation, stress-assisted oxidation, and ultimately loss of structural integrity, which are in turn accelerated by simultaneous hydrolytic or oxidative degradation [16,75,76]. This coupling is frequently synergistic: hydrolysis lowers molecular weight and embrittles the material, oxidation increases stiffness and reduces toughness by means of chain scission and crosslinking. All these phenomena, combined, will affect crack-growth behavior and notch sensitivity [16,77,78,79]. Conversely, mechanical damage (crazing, micro voids, delamination) increases surface area and permeability, facilitating deeper fluid ingress and amplifying chemical aging [73,80,81].

In summary, polymeric biomaterials provide a particularly informative case for dynamic biocompatibility because their long-term behavior emerges from the coupling of chemical degradation, interfacial bio-interactions, mechanical instability, and biological variability. The relevant biocompatibility endpoint is therefore not a fixed material property, but a system-level outcome that depends on time scale, functional context, and the evolving chemistry and mechanics of the implant–tissue interface. Here, we propose a conceptual framework for interpreting polymer degradation in biological environments as a directional network of coupled processes. Rather than attempting to define universal kinetic constants or a fully predictive degradation model, the framework is intended to organize mechanistic evidence, identify recurrent degradation archetypes, and guide the design of application-specific testing strategies.

## 2. The Biological Environment Surrounding Polymeric Implants

Any discussion of polymer degradation and bio-interaction must begin by defining the environment in which these materials operate. The biological environment surrounding a polymeric implant is an extraordinarily complex, dynamic system composed of water, ions, proteins, lipids, polysaccharides, reactive oxygen species (ROS), and a wide range of low-molecular weight metabolites [82,83].

From a thermodynamic standpoint, such an environment is never in true equilibrium. Its local composition fluctuates over time in response to metabolic activity, inflammation, healing, tissue remodeling and age [50,84]. Even within a single organism, two implants at different sites may experience radically distinct chemical conditions. For instance, the oxidative potential in synovial fluid differs substantially from that of blood plasma, and the concentration of enzymes capable of hydrolyzing ester bonds is significantly higher in inflamed tissues than in quiescent ones [85,86].

This variability makes it nearly impossible to define a single, universal “biological medium” representative of in vivo conditions. However, meaningful simplifications can still be made when describing the general environment surrounding polymeric implants, provided that their limitations are explicitly acknowledged [82,87].

The implant environment is aqueous and, in poorly vascularized regions, can be relatively oxygen-poor; this often makes hydrolytic and enzymatic degradation central pathways, although oxidative degradation may still become important under inflammatory ROS-rich conditions [29,88]. Ionic strength and pH remain within relatively narrow physiological ranges, but localized acidification can occur during polymer degradation and in inflammatory microenvironments [29,89].

Protein and lipid adsorption is effectively unavoidable after implantation. Adsorbed proteins can appear on biomaterial surfaces within seconds and form a conditioning layer within minutes, and this interfacial layer strongly influences the subsequent biological response [6,90,91]. Table 1 summarizes representative values for key environmental parameters across different anatomical locations commonly relevant to polymeric implants.

A common pitfall in biomedical polymer characterization arises from the use of overly simplified in vitro testing media [82,87]. While convenient and reproducible, these media often fail to replicate the full complexity of in vivo conditions [82,87]. In fact, what is being validated in many assays is not the material’s intrinsic stability, but its response to a very specific chemical boundary condition rarely found at the implant-tissue interface [82,87,104]. Phosphate-buffered saline (PBS), for example, is commonly used to study hydrolytic degradation phenomena [104,105]. However, it lacks proteins, lipids, and many low-molecular weight metabolites that influence surface interactions and enzymatic activity [87,106]. Similarly, fetal bovine serum (FBS), which is used as a source of proteins and growth factors, is not representative of the in vivo conditions because it is compositionally variable and can influence experimental outcomes in a batch-dependent manner [107,108]. Synovial fluid is particularly relevant in the study of orthopedic devices because it contains hyaluronic acid, lubricin, and specific enzymes not found in PBS or FBS [85,109]. It is therefore important that the testing environment is carefully chosen or is a combination of different solutions in order to obtain results that closely mimic the in vivo environment [82,87,110]. A pragmatic approach would be to adopt media that isolate mechanisms (pure hydrolysis in buffered salts, protein conditioning in defined protein mixtures, oxidative challenge with controlled ROS donors, enzymatic challenge with physiologically relevant esterases/lipases/proteases), and then combine them under realistic transport and mechanical conditions (media renewal, flow, cyclic loading).

Even if we would like to perfectly simulate the biological environment surrounding polymeric implants, testing must still follow established international standards to allow comparison and reproducibility of results. The ISO 10993 series provides the foundational framework for the biological evaluation of medical devices, including polymers—covering cytotoxicity (ISO 10993-5), degradation product identification (ISO 10993-13), and blood interaction (ISO 1099-34), among others [111]. ASTM standards such as F2027 and F2150 provide guidance on characterization and testing of raw biomaterials and scaffolds [112,113]. While highly valuable, none of these standards fully prescribe realistic physiological test media that mimic the full complexity surrounding an implant (e.g., water + ions + proteins + lipids + enzymes + ROS in dynamic equilibrium). Therefore, when researchers adopt simplified media (e.g., PBS, SBF, standard protein-supplemented buffer), they should explicitly note the gap to in vivo reality, and referencing existing standards is essential to allow meaningful comparison of results across different studies.

## 3. Mechanisms of Degradation for Biomedical Polymers

The performance and longevity of polymeric implants in vivo are governed not only by their initial mechanical and chemical properties but also by a variety of degradation mechanisms that progressively alter the polymer structure over time. Unlike metals and ceramics, whose in vivo degradation is typically limited to corrosion [114] or wear [115,116], polymers exhibit a diverse range of failure pathways, reflecting their structural versatility and chemical tunability. This diversity allows polymers to be better engineered for temporary scaffolds, drug delivery carriers, or permanent implants, but also introduces complex challenges in predicting long-term behavior.

Polymeric degradation is influenced by both intrinsic factors, such as chemical composition [117], crystallinity [118], molecular weight [119], and crosslink density [120], and extrinsic factors, including mechanical stresses [121], local biochemical environment [122], enzymatic activity [123], and exposure to reactive oxygen species [124]. In biological systems, multiple degradation pathways often operate simultaneously or sequentially, producing complex interactions that affect mechanical integrity, biocompatibility, and the release of degradation products. For example, a biodegradable PLA scaffold may simultaneously undergo hydrolysis, enzymatic cleavage, and surface oxidation, while the adsorption of proteins and lipids can locally modulate these processes.

**Figure 1:**
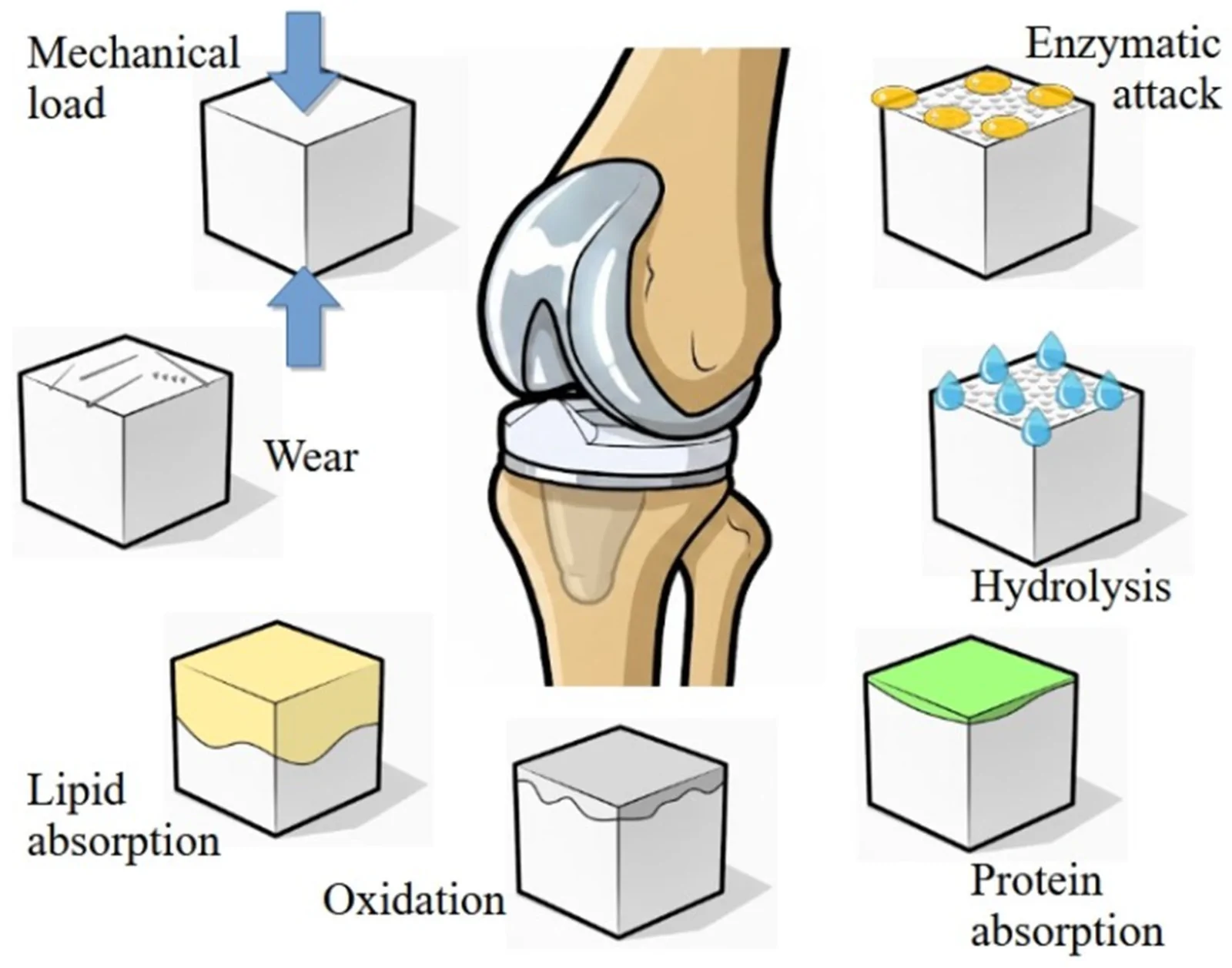
Schematic overview of the main degradation processes affecting polymeric biomaterials in vivo. Mechanical loading, wear, hydrolysis, oxidation, enzymatic attack, protein adsorption, and lipid absorption interact at the implant surface and within the material bulk.

A detailed understanding of these mechanisms is essential for the rational design of biomedical polymers, ensuring that their functional lifetime matches the intended clinical application, and that degradation products are non-toxic and bioresorbable when appropriate. As schematically illustrated in Figure 1 polymeric biomaterials are exposed in vivo to a combination of mechanical, chemical, and biological processes that can act simultaneously at the implant surface and within the material bulk. The interplay among these mechanisms may progressively alter the physicochemical and mechanical properties of the polymer, ultimately affecting its long-term performance and biostability. In the following sections, the major degradation mechanisms are discussed in detail, including mechanical fatigue, wear, lipid absorption, oxidation, protein adsorption, hydrolysis, and enzymatic degradation, with representative examples illustrating their relevance in real-world biomedical applications.

### 3.1. Mechanical Fatigue

Mechanical fatigue occurs when polymeric implants are subjected to repeated cyclic stresses that are below the material’s ultimate strength but sufficient to induce microstructural damage over time. In vivo, this is particularly relevant for load-bearing devices such as bone fixation plates [125], spinal rods [126], or joint prostheses [127], where repetitive physiological forces lead to microcracks that propagate and ultimately compromise mechanical integrity. For example, polycarbonate-urethane used in articulating surfaces of intervertebral disc replacements can develop microcracks under continuous flexion-extension cycles, potentially leading to implant failure even without chemical degradation [128]. Fatigue behavior is influenced by polymer crystallinity, molecular weight, and the presence of stress concentrators such as pores or surface defects.

### 3.2. Wear

Wear refers to the progressive loss of material from a polymer surface due to friction or sliding contact, often in combination with mechanical fatigue. Articulating implants, such as polyethylene components of total hip or knee replacements, are classic examples where wear generates debris particles that can trigger inflammatory responses, osteolysis, or implant loosening [129]. Crosslinked ultra-high molecular weight polyethylene (UHMWPE) has been engineered to improve wear resistance, but even minor deviations in surface finish or load distribution can accelerate degradation [130]. Wear is also critical for catheters and vascular grafts, where repeated flexing or contact with surrounding tissue can erode the polymer surface.

### 3.3. Lipid Absorption

Lipid absorption involves the penetration of hydrophobic molecules, such as fatty acids or cholesterol, into the polymer matrix [131]. This phenomenon can act as a plasticizer, softening the polymer and increasing its susceptibility to mechanical deformation or hydrolytic degradation [132]. For instance, poly(caprolactone) (PCL) and poly(lactic acid) (PLA) scaffolds exposed to lipid-rich environments, such as subcutaneous adipose tissue or lipid-laden synovial fluid, may exhibit accelerated creep or stress relaxation. Lipid uptake can also influence the release kinetics of embedded drugs in polymeric delivery systems by altering polymer chain mobility.

### 3.4. Oxidation

Oxidative degradation arises from the interaction of polymers with reactive oxygen species (ROS) or other oxidizing agents present in biological fluids [133]. In vivo, this mechanism is prominent in inflammatory environments, where macrophages and neutrophils generate ROS. Polymers containing susceptible groups, such as polyethers or polyurethanes, are prone to chain scission or crosslinking due to oxidative attack, which can reduce tensile strength and elasticity [134]. A practical example is polyurethane-based vascular grafts [135], which have been observed to undergo oxidative embrittlement over years of implantation, particularly near sites of chronic inflammation. Antioxidant additives or surface coatings can partially mitigate this process [136].

### 3.5. Protein Adsorption

Protein adsorption is one of the first events following implantation and profoundly affects the subsequent biological response and polymer degradation [137]. Within minutes, a conditioning film of serum proteins, including albumin, fibrinogen, and immunoglobulins, forms on the polymer surface [138]. Adsorbed proteins can mediate immune cell attachment, trigger enzymatic activity, or catalyze local hydrolysis. For example, collagen-coated poly(lactic-co-glycolic acid) (PLGA) scaffolds facilitate cell adhesion but also create localized regions where proteolytic enzymes accelerate degradation [139]. Protein adsorption is thus both a necessary component for bioactivity and a potential driver of heterogeneous polymer breakdown.

### 3.6. Hydrolysis

Hydrolytic degradation is the cleavage of hydrolysable bonds, such as esters, amides, or anhydrides, by water molecules [140]. This mechanism dominates the in vivo resorption of biodegradable polymers like PLA, PGA, PLGA, PDO and PCL [141]. Hydrolysis can be bulk or surface-limited depending on polymer crystallinity, molecular weight, and morphology. For example, PLGA nanoparticles designed for drug delivery gradually hydrolyze over days to weeks, releasing encapsulated therapeutics [142], while dense PLA screws used in orthopedic fixation may require months to fully resorb [143]. Hydrolytic rates are influenced by local pH, temperature, and water accessibility, often producing acidic byproducts that further autocatalyze degradation.

### 3.7. Enzymatic Degradation

Enzymatic degradation occurs when specific biological catalysts cleave polymer chains, often in a highly site-specific and regulated manner. Natural polymers such as collagen, gelatin, chitosan, and silk fibroin are particularly susceptible to proteases, lysozyme, or other hydrolases [144,145]. For instance, chitosan-based wound dressings are gradually broken down by lysozyme present in human serum, while silk fibroin scaffolds implanted in bone defects are resorbed over months via proteolytic cleavage. Enzymatic activity can also affect synthetic polymers modified with biodegradable linkages, such as ester [146] or peptide bonds, providing opportunities to design implants with controlled resorption in targeted tissues.

## 4. Conceptual Modeling of Degradation and Functionality Loss

Since degradation mechanisms interact, a simple additive model would be insufficient; instead, we can treat the overall degradation as a function that accounts for mutual interactions between mechanisms [147,148]. The following formalism is not intended as a predictive kinetic model. Instead, it provides a conceptual notation for distinguishing isolated degradation contributions from interaction-mediated functionality loss. All degradation variables should be understood as normalized, dimensionless descriptors defined with respect to a specific endpoint, such as molecular weight retention, mass retention, tensile-strength retention, fatigue-life reduction, or dielectric failure. Let $D\left(t\right)$ represent the overall degradation of a polymer at time t, which could be measured as mass loss, mechanical property loss, or chain scission fraction. In this framework, $D\left(t\right)$ should not be interpreted as a universal degradation variable that can be compared directly across different endpoints. Rather, $D\left(t\right)$ denotes an endpoint-specific, normalized descriptor of degradation, defined with respect to the functional property under consideration. For example, $D\left(t\right)$ may represent normalized mass loss, normalized molecular weight loss, normalized tensile-strength loss, reduction in fatigue life, loss of dielectric integrity, or another application-relevant degradation endpoint. Because these quantities have different physical meanings and units, they should not be summed unless they are first transformed into dimensionless variables on a common scale, typically ranging from 0, indicating no degradation relative to the initial state, to 1, indicating complete loss of the selected functional endpoint. Thus, every use of $D\left(t\right)$ requires explicit definition of the endpoint, normalization procedure, and failure threshold relevant to the intended biomedical application. We have n degradation mechanisms (e.g., hydrolysis, oxidation, mechanical fatigue, etc.). A simple additive model would be:

(1) $$D\left(t\right)=\sum_{i=1}^{n}M_{i}\left(t\right)$$

However, this ignores interactions between mechanisms. For a more realistic model, we can introduce interaction terms:

(2) $$D\left(t\right)=\sum_{i=1}M_{i}\left(t\right)+\sum_{i=1}\sum_{j>i}\alpha_{ij}M_{i}\left(t\right)M_{j}\left(t\right)$$

where $\alpha_{ij}$ is the interaction coefficient between mechanisms i and j, with $\alpha_{ij}>1$ for synergistic effects (accelerated degradation) and $\alpha_{ij}<1$ for antagonistic effects, where one mechanism slows the other.

This can be generalized to higher-order interactions, but in practice second-order interactions are often sufficient:

(3) $$D\left(t\right)=\sum_{i=1}M_{i}\left(t\right)+\sum_{i=1}\sum_{j>i}\alpha_{ij}M_{i}\left(t\right)M_{j}\left(t\right)\;\;\;\;\;\;\;\;\;\;\;\;\;\;\;\;\;\;\;\;\;\;\;\;\;\;\;\;\;\;\;\;\;\;\;\;\;\;\;\;\;\;\;\;\;\;\;\;\;\;\;\;\;\;\;\;+\sum_{i=1}\sum_{j>i}\sum_{k>j}\beta_{ijk}M_{i}\left(t\right)M_{j}\left(t\right)M_{k}\left(t\right)+\ldots$$

where $\beta_{ijk}$ is the second-order interaction coefficient between mechanisms i, j and k.

For the way $D\left(t\right)$ has been defined, it naturally appears as an increasing function, rising from zero toward a value that represents the accumulated effects of all degradation mechanisms. However, this abstract accumulation does not immediately convey the loss of polymer functionality, and it can be unintuitive, especially when comparing materials or predicting implant lifetime. For this reason, it is often more convenient and interpretable to define a functionality retention metric, $F\left(t\right)$, as the complement of $D\left(t\right)$:

(4) $$F\left(t\right)=1-D\left(t\right)$$

In this representation, $F\left(t\right)$ decreases monotonically from 1, corresponding to a fully functional polymer, to 0, indicating complete loss of mechanical, chemical, or biological functionality. This approach aligns directly with experimentally measurable quantities, such as tensile strength, mass retention, or molecular weight, allowing for a more intuitive visualization of degradation kinetics. Moreover, $F\left(t\right)$ provides a clear framework for comparing polymers, formulations, or environmental conditions, highlighting that different mechanisms may contribute unevenly to the overall functional decline and that mass loss, molecular damage, and mechanical deterioration are often not perfectly synchronous.

In order to use $F\left(t\right)$ meaningfully, it is essential to define what is meant by polymer functionality. In biomedical applications, functionality is typically related to mechanical performance or structural integrity, depending on the intended use of the implant.

- *Mechanical Functionality*: For load-bearing implants such as bone fixation screws or intervertebral disc scaffolds, polymer functionality is directly linked to mechanical properties like tensile strength, elastic modulus, and fatigue resistance [149,150,151]. Importantly, mechanical functionality does not require complete loss of strength to indicate degradation; it is lost when the polymer can no longer meet the specific load-bearing requirements of its intended application [152,153]. This can be formalized as follows:

(5) $$F_{mech}\left(t\right)=\frac{\sigma \left(t\right)-\sigma_{min}}{\sigma_{0}-\sigma_{min}}$$

where $\sigma_{0}$ is the initial strength, $\sigma_{min}$ is the minimum required strength to fulfill the role and $\sigma \left(t\right)$ is the tensile strength at time t;

- *Structural or Mass Retention*: For resorbable scaffolds, sutures, or non-load-bearing implants, functionality can be expressed in terms of mass retention or dimensional stability, reflecting the polymer’s ability to maintain shape and provide mechanical support [153,154,155]. Here, $F_{mass}\left(t\right)$ tracks how much of the original polymer remains before it is completely resorbed [156,157].

By defining $F\left(t\right)$ in terms of a specific functional metric, whether mechanical or structural, it becomes possible to link the abstract concept of degradation to measurable outcomes, enabling systematic comparison of different polymers, formulations, or environmental conditions. This also provides a clear basis for incorporating the contributions of multiple interacting degradation mechanisms, as discussed in the previous sections.

It is also important to note that multiple definitions of functionality can be applied simultaneously, depending on the intended role of the polymeric implant. For example, in a bioresorbable scaffold, mechanical functionality must be maintained for a specific period to provide structural support during tissue regeneration [143,158]. During this phase, $F_{mech}\left(t\right)$ is the critical metric, and degradation is monitored with respect to the retention of sufficient mechanical strength. Once the tissue has matured and the scaffold’s structural role is no longer required, the degradation process can continue primarily in terms of mass loss, captured by $F_{mass}\left(t\right)$, to ensure complete resorption without leaving harmful residues [159].

In this way, $F_{mech}\left(t\right)$ and $F_{mass}\left(t\right)$ provide complementary perspectives: the former governs functionality while support is needed, and the latter describes the subsequent clearance of the polymer. The combined approach allows for a more accurate and physiologically relevant assessment of degradation kinetics in biomedical polymers, highlighting the time-dependent relationship between functional performance and structural resorption.

## 5. Degradation Interaction Matrix

In this framework, however, it becomes clear that the interaction coefficients $\alpha_{ij}$ and, by extension, the higher-order terms such as $\beta_{ijk}$, cannot currently be quantified with any realistic degree of confidence. Their determination would require detailed knowledge of coupled reaction kinetics, local microenvironmental dynamics, and time-dependent feedback processes, a level of resolution that neither experimental models nor computational approaches can yet provide. Thus, although these coefficients represent the most rigorous way to describe synergistic or antagonistic interactions, they remain fundamentally inaccessible at present.

For this reason, it is necessary to introduce an intermediate level of abstraction that allows us to capture at least the directionality of the dependencies between degradation mechanisms, even when their magnitude remains unknown.

The interaction matrix presented in Table 2 conceptualizes polymer degradation as a directed amplification network rather than a set of independent or mutually symmetric processes. Interaction strengths were assigned according to three qualitative criteria: mechanistic plausibility, recurrence across more than one polymer family or biomedical context, and evidence that activation of the upstream mechanism changes the rate, spatial extent, or functional consequence of the downstream mechanism. “Negligible interaction” indicates no consistent mechanistic or experimental support for amplification under physiological conditions; “weak facilitation” indicates plausible or context-dependent enhancement; and “strong synergistic amplification” indicates recurrent evidence that one mechanism substantially increases susceptibility to another. These scores should not be interpreted as material constants, but as qualitative descriptors intended to organize mechanistic evidence and generate testable hypotheses for specific polymer–environment–device combinations. Each matrix element $I_{ij}$ qualitatively represents the extent to which mechanism i exacerbates mechanism j, acknowledging that $I_{ij}\neq I_{ji}$ in most cases. This asymmetry reflects the inherently directional nature of degradation pathways. For instance, hydrolysis-induced molecular weight reduction substantially increases susceptibility to mechanical fatigue [160], whereas mechanical fatigue enhances hydrolytic degradation primarily through secondary effects such as microcrack-mediated water ingress [161]. Similarly, protein adsorption may locally concentrate enzymes and strongly promote enzymatic degradation [161], while the reverse interaction is comparatively limited.

Such directionality underscores that degradation mechanisms do not operate in parallel but form a causal network with identifiable hubs and feedback loops. Hydrolysis and oxidation emerge as central amplifiers due to their capacity to alter polymer chemistry and mechanical integrity at a molecular level [133,162], thereby increasing vulnerability to secondary processes such as fatigue and wear. Mechanical fatigue and wear, in turn, act as mediators that expose fresh reactive surfaces and accelerate chemical degradation pathways.

Importantly, the interaction strengths reported are qualitative and derived from established mechanistic evidence across polymeric biomaterials. The magnitude and even the sign of specific interactions remain dependent on polymer chemistry, morphology, and local physiological conditions [14]. Nonetheless, this directed framework provides a structured basis for incorporating interaction terms into mechanistic degradation models and supports the view that long-term implant performance is governed by coupled, non-linear amplification processes rather than additive damage accumulation. Interaction strengths are derived from established mechanistic evidence across polymer systems and are intended to provide a conceptual framework; quantitative magnitudes are polymer- and environment-dependent.

## 6. Categories of Polymers Used in Biomedical Applications

**Figure 2:**
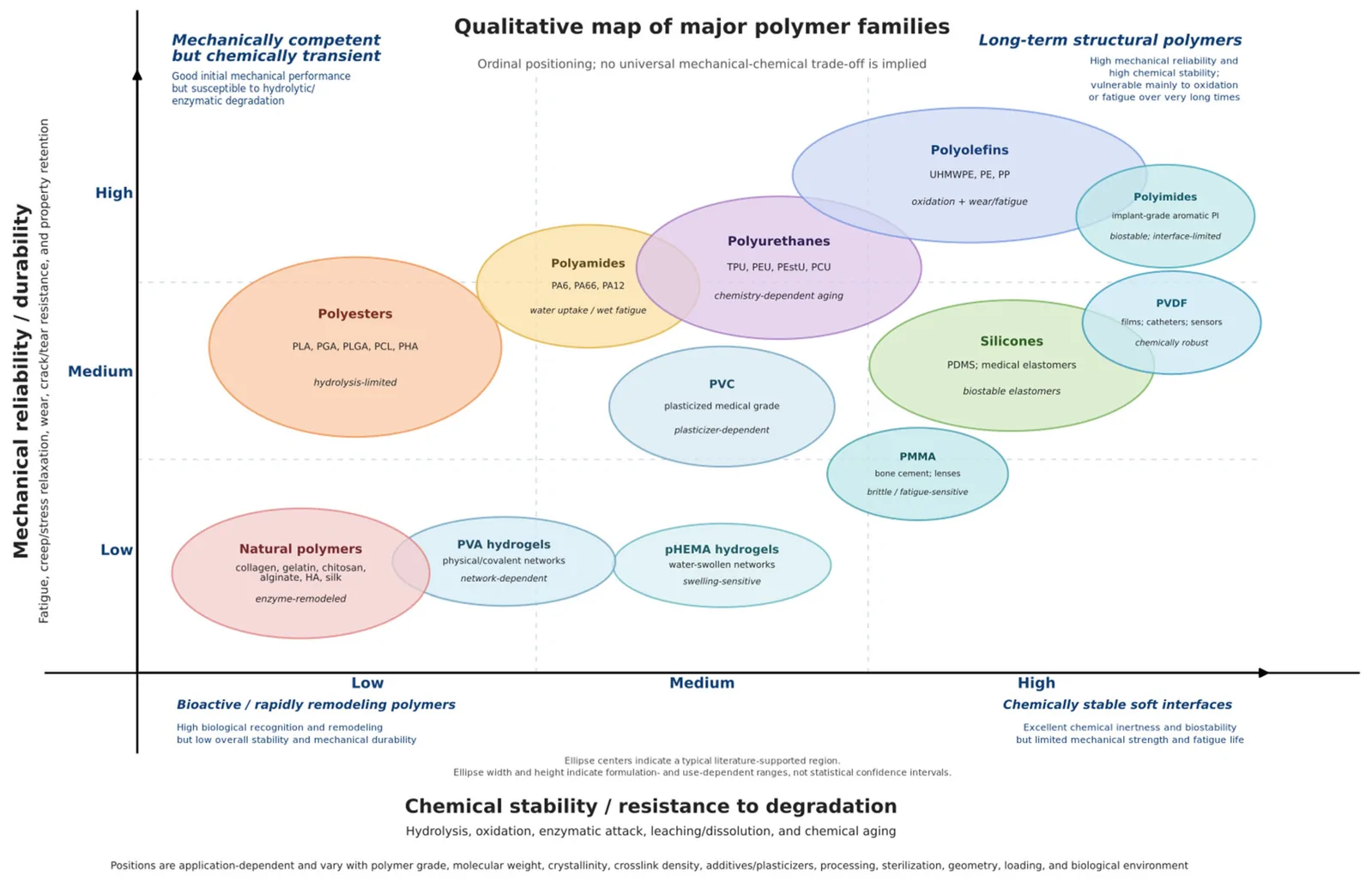
Qualitative positioning of major polymer families according to mechanical reliability/durability and chemical stability/resistance to degradation.

Compared with metals and ceramics, which are primarily valued for their high mechanical strength, wear resistance, and chemical stability, polymers offer an extraordinary degree of tunability. Their mechanical properties, degradation rates, hydrophilicity, and surface chemistry can be tailored by altering monomer composition [163], copolymer ratios [164], molecular weight [165], or by adding functional groups [166]. Beyond chemistry, architecture and processing are as important: crystallinity [167], tacticity [168], crosslink density [169], and chain orientation [170] can be modulated through thermal history, annealing, stretching, and additive manufacturing, while composites (fibers, nanoparticles, porous reinforcements) enable further control over stiffness, toughness, and permeability [171]. Equally important, polymer interfaces can be engineered independently of the bulk via surface grafting [172], plasma treatments [173], coatings [174], or immobilization of peptides [175] and polysaccharides [176], allowing selective tuning of protein adsorption [177], cell adhesion [178], antifouling behavior [179], and antimicrobial activity [180] without sacrificing structural requirements. This high specialization enables applications ranging from load-bearing temporary bone fixation devices to soft tissue scaffolds, drug delivery carriers, and flexible implants, all within the same chemical family. The map in Figure 2 compares representative biomedical and engineering polymers along two conceptual axes: mechanical reliability and durability, including fatigue resistance, creep resistance, wear tolerance, crack-propagation resistance, and environmental resistance, versus chemical stability, including resistance to hydrolysis, oxidation, enzymatic attack, and chemical aging. Elliptical regions indicate typical performance ranges for each polymer family, while annotations summarize key degradation mechanisms and limiting factors. The four corner regions identify broad application domains: bioactive or rapidly remodeling polymers, mechanically competent but chemically transient polymers, chemically stable soft interfaces, and long-term structural polymers. The positioning of each material class was established by comparing literature-reported trends in fatigue and wear resistance, mechanical stability, and resistance to crack propagation along the mechanical axis, and in hydrolytic, oxidative, enzymatic, and chemical stability along the chemical axis. Polymer families were placed according to their typical behavior rather than that of individual optimized grades. Accordingly, the resulting map should be interpreted as a qualitative framework rather than as a quantitative ranking.

For example, polyesters such as PLA, PGA, and PLGA exhibit hydrolytic degradation rates that can be tuned from weeks to months depending on the monomer ratio and crystallinity, allowing precise control over resorption in vivo [181]. In contrast, polyolefins like PE and PP are essentially inert and non-degradable, making them suitable for long-term structural applications such as vascular grafts and surgical meshes [182]. Here, long-term performance is less limited by hydrolysis than by physical aging and oxidative phenomena. In vivo oxidation (often intensified under inflammatory ROS exposure) can lead to embrittlement, surface cracking, and altered wear behavior even in polymers considered “chemically stable”. Thus, stabilization strategies (antioxidants, crosslinking, coatings) and the local inflammatory response can become decisive determinants of lifetime.

Hydrophilic polymers like PVA and pHEMA form water-swollen hydrogels suitable for cell encapsulation or contact lenses [183], while elastomeric silicones and polyurethanes provide flexibility and durability for catheters and heart valves. In hydrogels, water content and network architecture govern not only softness and diffusion (nutrient/drug transport) but also susceptibility to mechanical fatigue under cyclic deformation. Subtle changes in crosslink density can therefore shift both biological performance and tear resistance. For silicones and polyurethanes, the clinical advantage of elasticity is balanced by environment-dependent aging: silicones may be affected by lipid absorption and surface fouling, whereas polyurethanes can undergo oxidative degradation (and, in susceptible chemistries, hydrolysis or environmental stress cracking), with property changes that emerge over long implantation times. These examples underline a broader point: polymer classes are best compared not only by “degradable vs. non-degradable” labels, but by their dominant in vivo aging modes (hydrolysis, oxidation, enzymatic attack, physical aging) and by how processing and morphology steer those modes in specific device contexts.

The variability in polymer properties is further amplified by the possibility of forming copolymers, blends [184], or composites [185], incorporating bioactive molecules (e.g., hydroxyapatite, growth factors, antioxidants), or by functionalizing surfaces to modulate cell adhesion and protein adsorption [186]. This combinatorial flexibility contrasts sharply with metals and ceramics, where properties are primarily dictated by crystal structure and composition, and tuning often requires alloying or surface coatings.

Given this huge range of chemical structures and tunable properties, it is useful to classify biomedical polymers according to their backbone chemistry, which largely governs mechanical behavior, degradation pathways, and biocompatibility [187,188,189]. The following sections summarize the major polymer classes used in biomedical applications, highlighting representative polymers, their chemical structures, typical applications, and key properties.

### 6.1. Polyesters

Aliphatic polyesters are characterized by hydrolytically labile ester bonds within the polymer backbone. The carbonyl carbon in the –COO– linkage is susceptible to nucleophilic attack by water, resulting in random chain scission and progressive molecular weight reduction. In bulk-eroding systems such as PLA and PLGA, hydrolysis initially reduces chain length without immediate mass loss, but progressively compromises mechanical integrity as entanglement density decreases [190].

**Figure 3:**
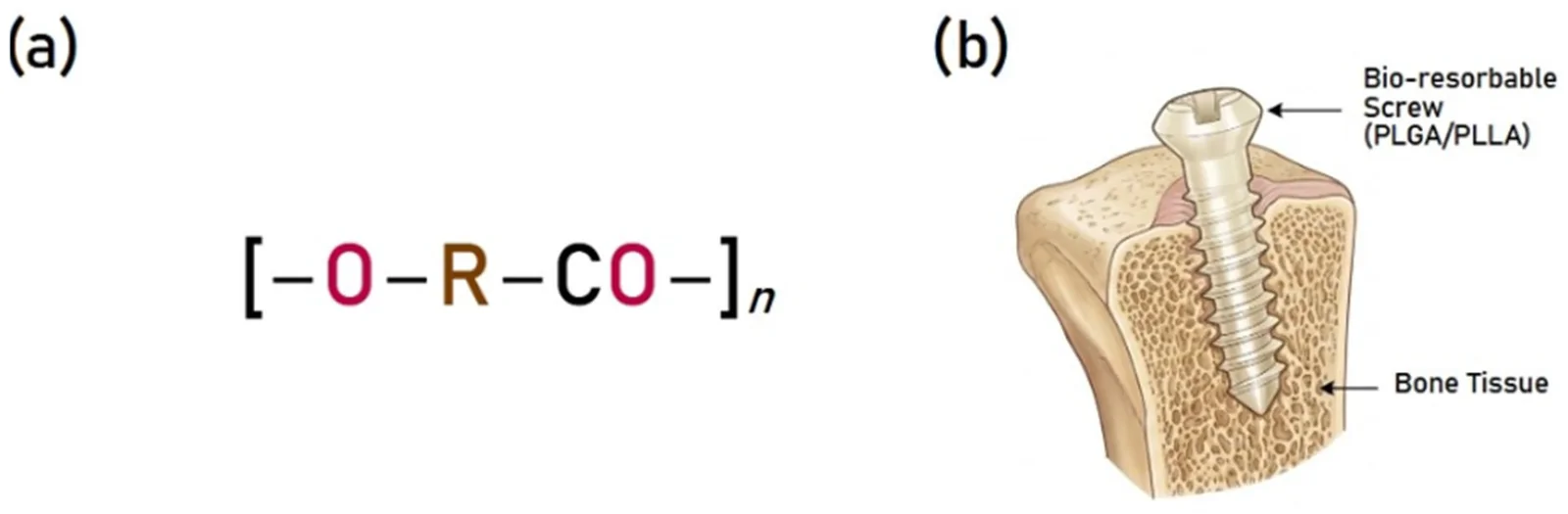
(**a**) Generic repeating structural unit of aliphatic polyesters, highlighting the ester linkage responsible for hydrolytic degradability. (**b**) Example of a typical biomedical application: a bioresorbable fixation screw used in orthopedic surgery.

Figure 3 summarizes the structure–application relationship of aliphatic polyesters. The hydrolytically labile ester linkage within the polymer backbone (Figure 3a) represents the key structural feature governing their degradation, whereas the bioresorbable orthopedic fixation screw shown in Figure 3b exemplifies how this controlled degradation can be exploited in temporary load-bearing biomedical devices.

Within the degradation network framework proposed in this review, hydrolysis acts as the primary initiating mechanism and central amplification hub for this polymer class. Molecular weight reduction directly increases susceptibility to mechanical fatigue by lowering crack initiation thresholds and facilitating crack propagation. Mechanical damage, including microcrack formation, enhances fluid ingress and may promote localized accumulation of acidic degradation products, accelerating further hydrolytic cleavage through autocatalytic effects. Surface wear can additionally expose fresh reactive regions, while oxidative reactions may occur secondary to inflammation-driven reactive species, further destabilizing the polymer matrix [191].

The resulting degradation process is therefore not purely chemical but emerges from a coupled chemical–mechanical amplification cascade. Clinically, this manifests as bulk erosion accompanied by progressive embrittlement and, in load-bearing contexts, accelerated fatigue fracture. Material design strategies aimed at interrupting this loop include increasing crystallinity, modifying copolymer composition, end-group stabilization, and incorporation of buffering or antioxidant components.

*Typical biomedical applications:*

- Resorbable sutures [192]
- Orthopedic fixation devices (screws, pins, plates) [193]
- Tissue engineering scaffolds [194]
- Drug delivery systems [195]

*Chemistry:* Ester backbone (–COO–)

*Intrinsic vulnerability:* Hydrolysis (Hy) dominant [104]

*Network role:* Hydrolysis = primary degradation hub

*Failure phenotype:*

- Bulk erosion [104]
- Embrittlement [196]
- Accelerated fatigue fracture [197]

### 6.2. Polyolefins

Polyolefins are characterized by chemically stable, saturated carbon–carbon backbones that are inherently resistant to hydrolysis and enzymatic attack [198]. Their widespread use in load-bearing biomedical applications, particularly UHMWPE in orthopedic articulating components, derives from their high toughness, low friction coefficient, and favorable biocompatibility [199].

**Figure 4:**
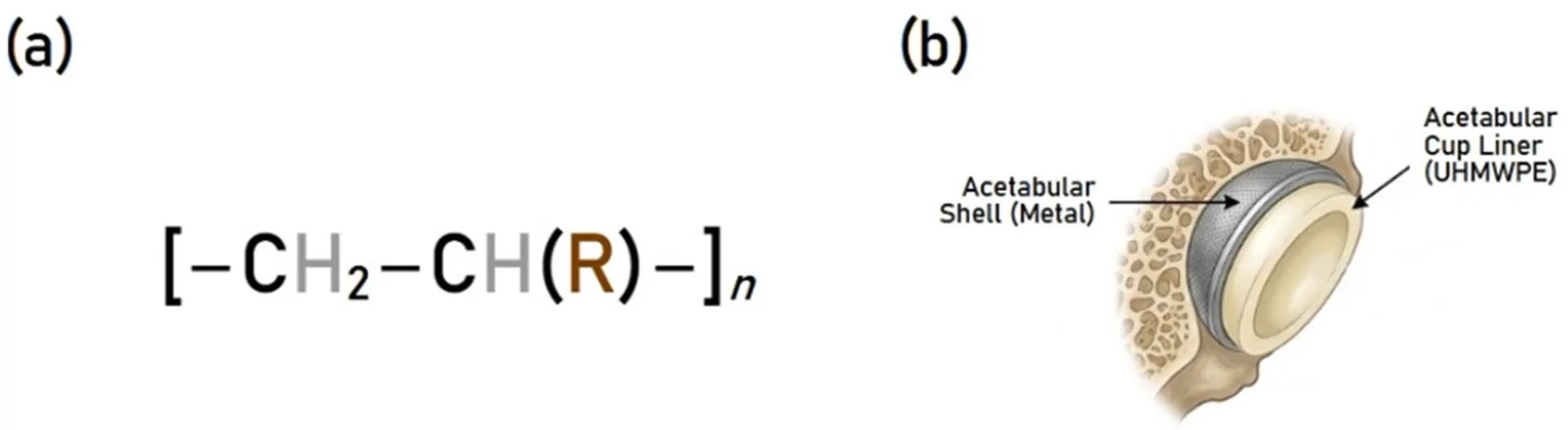
(**a**) Generic repeating structural unit of polyolefins, consisting of a saturated hydrocarbon backbone. (**b**) Example of a typical biomedical application: an UHMWPE liner used in the acetabular cup of total hip replacements.

As illustrated in Figure 4, the saturated hydrocarbon backbone of polyolefins (Figure 4a) provides high chemical stability and resistance to hydrolysis, supporting their use in demanding load-bearing applications. A representative example is the UHMWPE acetabular cup liner shown in Figure 4b, widely employed in total hip arthroplasty because of its favorable toughness, low friction, and wear performance.

Despite their chemical stability under aqueous conditions, polyolefins are susceptible to oxidative degradation. Oxidation may be initiated during sterilization (e.g., gamma irradiation) or occur in vivo due to reactive oxygen species generated by inflammatory cells. Oxidative reactions introduce carbonyl species and induce chain scission, leading to molecular weight reduction and microstructural changes such as crystallinity alterations. These chemical modifications progressively reduce ductility and fracture resistance [200].

Within the proposed degradation network, oxidation functions as the primary hub mechanism. Oxidative chain scission results in embrittlement, which significantly increases susceptibility to mechanical fatigue and wear under cyclic loading. Wear processes expose fresh material surfaces and may disrupt protective oxidized layers, thereby facilitating further oxygen diffusion and continued oxidative attack. This creates a chemically initiated but mechanically amplified feedback loop, particularly relevant in articulating joint replacements where repetitive motion and contact stresses dominate.

Clinically, this coupled oxidation–wear network manifests as surface delamination, generation of wear debris, and long-term fatigue-assisted cracking. The biological response to wear particles may further sustain oxidative conditions, indirectly reinforcing the degradation cycle. Material strategies aimed at interrupting this loop include antioxidant stabilization (e.g., vitamin E incorporation), improved crosslinking protocols, and optimized sterilization methods to minimize residual free radicals.

*Typical biomedical applications:*

- Orthopedic bearing components (hip and knee joint replacements) [200]
- Spinal cages [200]
- Hernia meshes [201]
- Surgical implants and tubing [202]

*Chemistry:* Saturated carbon–carbon backbone (–C–C–), absence of hydrolytically labile bonds

*Intrinsic vulnerability:* Oxidation (Ox) dominant [203]

*Network role:* Oxidation = primary degradation hub

*Failure phenotype:*

- Surface delamination [35]
- Wear debris generation [204]
- Oxidative embrittlement [205]
- Fatigue-assisted cracking [206]

### 6.3. Polyamides

Polyamides contain amide bonds (–CONH–) within the polymer backbone, which are stabilized by extensive intermolecular hydrogen bonding. This structural feature imparts relatively high mechanical strength and semicrystalline morphology but also introduces sensitivity to moisture [207]. While amide bonds are more resistant to hydrolysis than ester linkages, prolonged exposure to aqueous or physiological environments can lead to slow chain scission, particularly under elevated temperature or mechanical stress.

**Figure 5:**
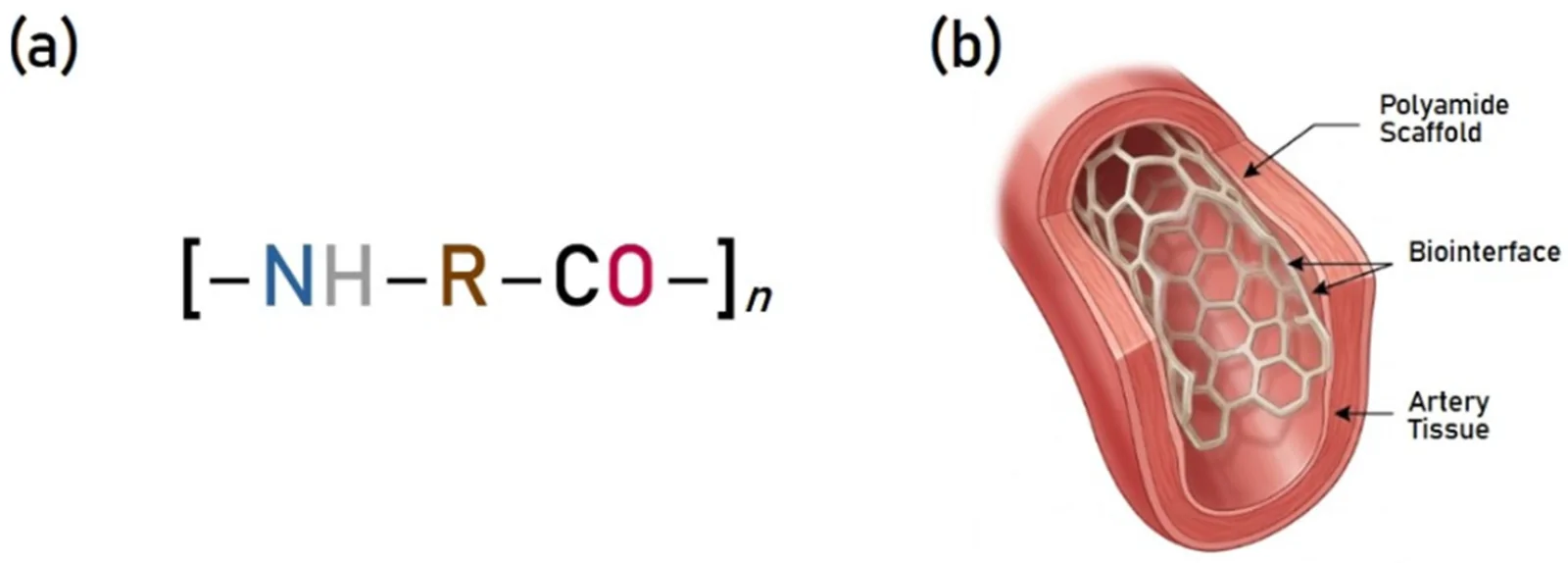
(**a**) Generic repeating structural unit of polyamides, characterized by amide linkages (–CONH–) along the polymer backbone. (**b**) Example of a biomedical application: a polyamide-based inter-arterial scaffold used for vascular support.

As shown in Figure 5, the amide-containing backbone of polyamides (Figure 5a) provides strong intermolecular hydrogen bonding and favorable mechanical properties, while also contributing to their moisture sensitivity. Figure 5b shows a representative polyamide-based vascular scaffold, illustrating the use of this polymer class in mechanically supportive biomedical applications exposed to physiological environments.

A defining characteristic of polyamides is their tendency to absorb water, which disrupts hydrogen bonding and induces plasticization. This water uptake increases chain mobility, reduces modulus, and promotes time-dependent deformation (creep). In contrast to polyesters, hydrolysis in polyamides is typically slower and less dominant; however, the combination of moisture-induced plasticization and cyclic mechanical loading can significantly accelerate fatigue processes [208].

Within the degradation network framework, polyamides are best described as coupled hydrolysis–mechanical systems. Hydrolytic chain scission gradually reduces molecular weight and mechanical strength, increasing susceptibility to crack initiation. Mechanical fatigue generates microstructural damage that enhances fluid ingress, locally facilitating further hydrolytic attack. At the same time, water absorption alone can increase creep deformation, indirectly amplifying mechanical degradation even in the absence of significant chemical scission.

Clinically, this degradation phenotype manifests as gradual loss of stiffness, creep deformation under sustained load, and fatigue-assisted cracking in long-term moist environments. Design strategies focus on increasing crystallinity, optimizing molecular weight, and reducing water uptake through copolymerization or surface modification to interrupt the moisture–mechanical amplification loop.

*Typical biomedical applications:*

- Surgical sutures [209]
- Catheters and tubing [210]
- Orthopedic components [211]
- Dental devices [212]
- Flexible implantable parts [213]

*Chemistry:* Amide linkages (–CONH–) in the backbone with hydrogen-bonded crystalline domains

*Intrinsic vulnerability:* Hydrolysis (Hy) moderate; water absorption–induced plasticization [208]

*Network role:* Coupled hydrolysis–mechanical system (Hy–Me interaction dominant)

*Failure phenotype:*

- Creep deformation [214]
- Fatigue-assisted cracking [215]
- Gradual embrittlement in moist environments [215]

### 6.4. Polyurethanes

**Figure 6:**
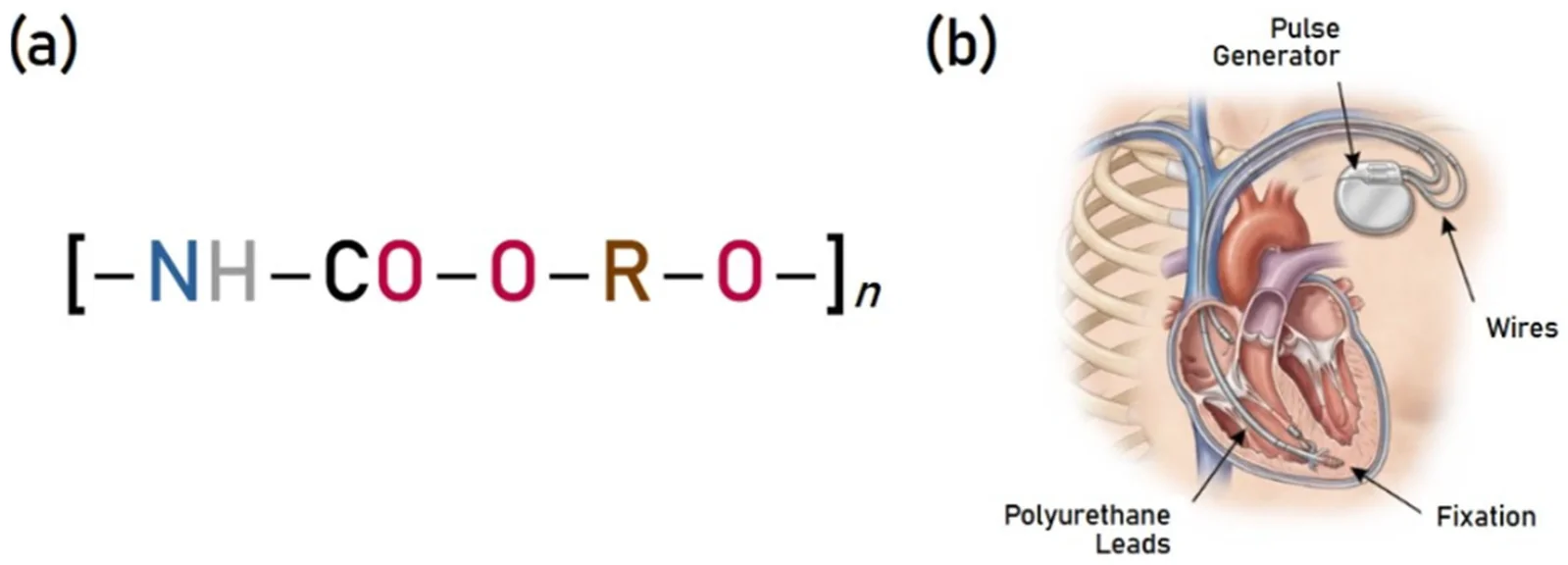
(**a**) Generic repeating structural unit of polyurethanes, characterized by urethane linkages (–NH–COO–) within the polymer backbone. (**b**) Example of a biomedical application: polyurethane insulation used in pacemaker leads.

Polyurethanes are segmented polymers composed of alternating soft segments (typically polyether or polyester chains) and hard segments formed by diisocyanate–chain extender reactions. This phase-separated morphology provides a combination of elasticity, toughness, and processability, making polyurethanes widely used in flexible, load-bearing, and blood-contacting biomedical devices [216]. As depicted in Figure 6, the urethane-containing backbone of polyurethanes (Figure 6a), together with their segmented soft- and hard-phase architecture, provides the combination of flexibility and mechanical strength required for demanding biomedical applications. Figure 6b shows a representative example of polyurethane insulation in pacemaker leads, where long-term mechanical durability and electrical insulation are essential for device performance.

Unlike polyolefins, polyurethanes contain chemically reactive functional groups that are susceptible to both oxidative and enzymatic attack [217,218]. Oxidative degradation, often mediated by reactive oxygen species released by inflammatory cells, can induce chain scission in both soft and hard segments. In polyether-based systems, oxidative attack on ether linkages is particularly relevant, whereas polyester-based polyurethanes may additionally exhibit hydrolytic sensitivity [219]. Enzymatic degradation, especially in vivo, may target specific segments depending on chemical composition and local biological activity.

Within the degradation network framework, polyurethanes behave as dual-hub systems in which oxidation and enzymatic processes act as primary initiators. Oxidative chain scission disrupts phase morphology and reduces molecular weight, leading to embrittlement and increased susceptibility to mechanical fatigue. Microcrack formation enhances fluid penetration and reactive species diffusion, reinforcing oxidative attack in a positive feedback loop. Simultaneously, protein adsorption on device surfaces may concentrate enzymes locally, promoting enzymatic cleavage and further weakening the polymer matrix. These processes are strongly asymmetric: protein adsorption can markedly enhance enzymatic degradation, whereas enzymatic scission does not equivalently increase adsorption [220].

Clinically, this coupled network behavior manifests as environmental stress cracking, surface pitting, progressive mechanical weakening, and in some cardiovascular applications, calcification associated with oxidative and inflammatory processes. Strategies to mitigate degradation include modification of soft-segment chemistry (e.g., polycarbonate-based systems), incorporation of antioxidant stabilizers, and surface treatments to reduce protein adsorption and inflammatory activation.

*Typical biomedical applications:*

- Cardiovascular devices (catheters, pacemaker leads, vascular grafts) [221]
- Blood-contacting tubing [222]
- Implantable coatings [223]
- Soft tissue substitutes [224]

*Chemistry:* Urethane linkages (–NH–CO–O–) with phase-separated soft and hard segments

*Intrinsic vulnerability:* Oxidation (Ox) and enzymatic degradation (En) [217,218]

*Network role:* Dual-hub system (Ox + En) with strong chemical–mechanical coupling

*Failure phenotype:*

- Environmental stress cracking [225]
- Surface oxidation and pitting [226]
- Calcification (in cardiovascular environments) [227]
- Fatigue-assisted rupture [206]

### 6.5. Silicones/Organosiloxanes

**Figure 7:**
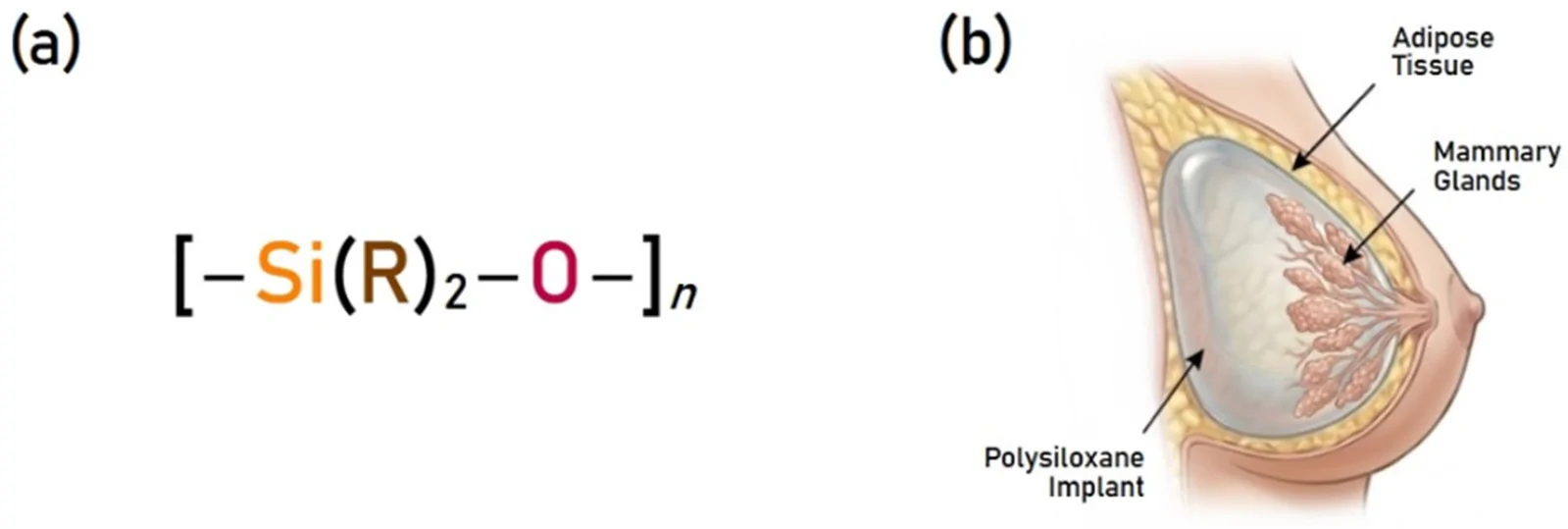
(**a**) Generic repeating structural unit of polysiloxanes, characterized by the –Si–O–Si– backbone typical of silicone polymers. (**b**) Example of a biomedical application: a silicone breast implant used in reconstructive and cosmetic surgery.

Silicones are characterized by a siloxane backbone (–Si–O–Si–), which provides exceptional flexibility due to the high bond angle and rotational freedom of the Si–O linkage. This structural feature results in low glass transition temperatures and high elasticity, making silicone elastomers particularly suitable for soft, flexible biomedical applications. The siloxane backbone of silicones (Figure 7a) provides high chain flexibility and elasticity, supporting their use in soft and deformable biomedical devices. Figure 7b shows a representative silicone breast implant, where long-term flexibility, biocompatibility, and resistance to physiological environments are essential for implant performance.

Unlike many carbon-based polymers, silicones are relatively resistant to hydrolysis [228] and enzymatic degradation [229] under physiological conditions. The Si–O bond is stable in neutral aqueous environments, and the hydrophobic methyl side groups limit water uptake. As a result, chemical degradation mechanisms such as hydrolysis play a minor role in most biomedical settings.

However, silicones are mechanically vulnerable under long-term cyclic deformation. Repeated loading can lead to microcrack initiation, particularly at defects, inclusions, or stress concentrators [230]. Surface damage may facilitate limited oxidative reactions, especially in inflammatory environments, potentially altering crosslink density locally. Although oxidation is generally secondary to carbon-based polymers, localized chemical modifications can increase stiffness heterogeneity and promote further crack propagation.

Within the degradation network framework, silicones are best described as mechanically driven systems in which fatigue acts as the primary initiating mechanism. Chemical processes may occur as secondary amplifiers but do not typically constitute dominant hubs. The resulting degradation phenotype is therefore governed primarily by fatigue crack growth and tear propagation rather than bulk chemical instability.

Clinically, failure often manifests as slow crack growth leading to rupture in long-term implants subjected to cyclic deformation. Design strategies focus on improving tear resistance, optimizing crosslink density, minimizing defect populations, and reinforcing elastomer matrices to interrupt mechanically driven amplification pathways.

*Typical biomedical applications:*

- Breast implants [231]
- Soft tissue implants [232]
- Catheters and drains [233]
- Shunts and flexible tubing [234]
- Encapsulation of electronic implants [235]

*Chemistry:* Siloxane backbone (–Si–O–Si–) with organic side groups (typically methyl)

*Intrinsic vulnerability:* Mechanical fatigue (Me) dominant; surface oxidation secondary [236]

*Network role:* Mechanically driven system with limited chemical amplification

*Failure phenotype:*

- Fatigue crack propagation [237]
- Oxidation [238]

### 6.6. Polyacrylates/Methacrylates

**Figure 8:**
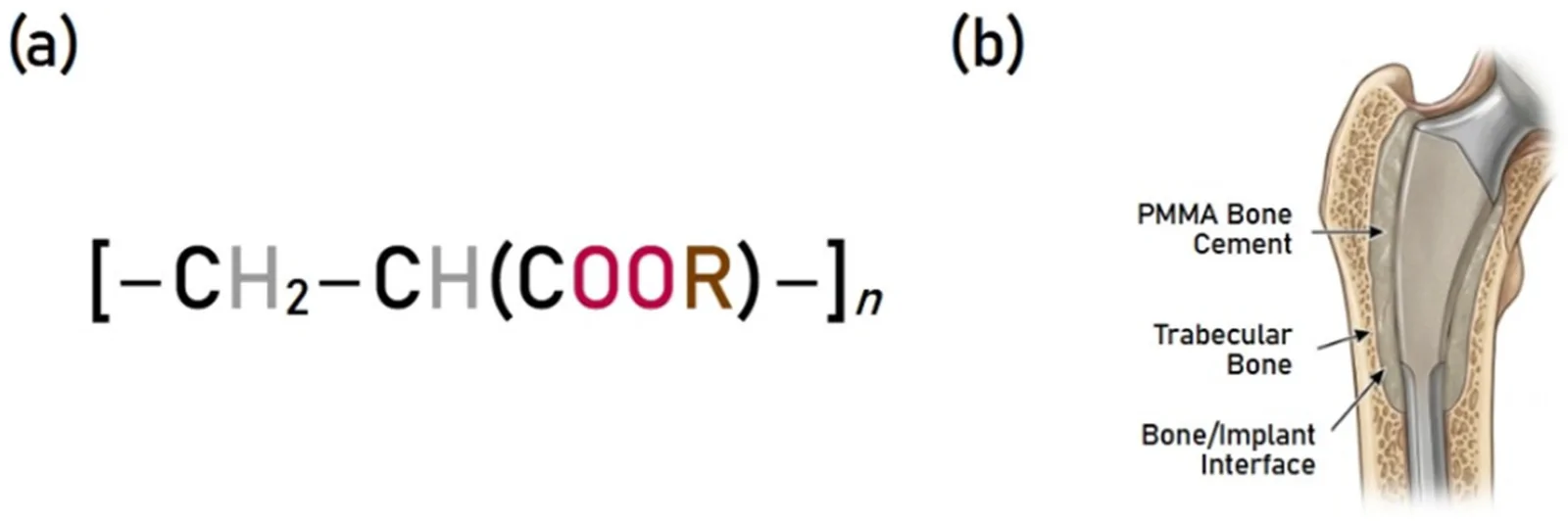
(**a**) Generic repeating structural unit of polyacrylates and polymethacrylates, characterized by a vinyl backbone bearing ester side groups. (**b**) Example of a biomedical application: poly(methyl methacrylate) (PMMA) bone cement used for fixation in orthopedic surgery.

Polyacrylates and methacrylates are characterized by a carbon–carbon backbone derived from vinyl polymerization, with pendant ester groups attached to the main chain. This architecture confers good optical clarity, stiffness, and processability, explaining their extensive use in bone cements, ophthalmic devices, dental materials, and hydrogel systems. As illustrated in Figure 8, polyacrylates and methacrylates possess a saturated carbon–carbon backbone bearing ester-containing side groups (Figure 8a), a structural arrangement that combines chemical stability with versatile mechanical and processing properties. Figure 8b shows a representative application of PMMA as bone cement for orthopedic fixation, where stiffness, dimensional stability, and load transfer are essential for implant performance.

Because the polymer backbone consists of saturated C–C bonds, these materials are generally resistant to backbone hydrolysis. However, the ester-containing side groups can undergo hydrolytic cleavage under prolonged exposure to moisture, particularly in hydrophilic variants such as PHEMA [239]. Side chain hydrolysis may alter polarity and plasticization behavior without necessarily inducing immediate backbone scission. In parallel, oxidative processes—especially under inflammatory conditions or UV exposure—can induce chain scission, crosslinking alterations, and carbonyl formation, progressively reducing fracture toughness.

Within the degradation network framework, polyacrylates are best described as oxidation-initiated systems [240] with significant mechanical amplification. Oxidative embrittlement lowers resistance to crack initiation and promotes crazing phenomena under tensile stress. Mechanical loading then accelerates crack growth, exposing fresh polymer surfaces to oxygen and aqueous media, reinforcing oxidative and hydrolytic processes. In hydrophilic methacrylates, water uptake further increases chain mobility and susceptibility to stress-induced damage [241], creating a coupled plasticization–fatigue loop.

Clinically, degradation often manifests as environmental stress cracking, surface crazing, or progressive embrittlement under cyclic loading. In bone cement applications [242], fatigue-assisted fracture and microcrack accumulation are particularly relevant, whereas in hydrogel-based systems [243], long-term stability may be limited by swelling-induced mechanical weakening. Material strategies to mitigate these effects include copolymer design to reduce residual stresses, antioxidant incorporation, optimization of crosslink density, and surface treatments to limit oxidative exposure.

*Typical biomedical applications:*

- Bone cement (PMMA) [244]
- Intraocular lenses [245]
- Denture bases and dental composites [246]
- Contact lenses (hydrogel-based methacrylates) [247]
- Injectable fillers and coatings [248]

*Chemistry:* Carbon–carbon backbone with ester-containing side groups (–COOR)

*Intrinsic vulnerability:* Oxidation (Ox) and side chain hydrolysis (Hy, secondary); environmental stress cracking [249]

*Network role:* Oxidation-initiated system with strong mechanical coupling

*Failure phenotype:*

- Environmental stress cracking [249]
- Surface crazing [250]
- Embrittlement [249]
- Fatigue-assisted fracture [249]

### 6.7. Polyvinyls

**Figure 9:**
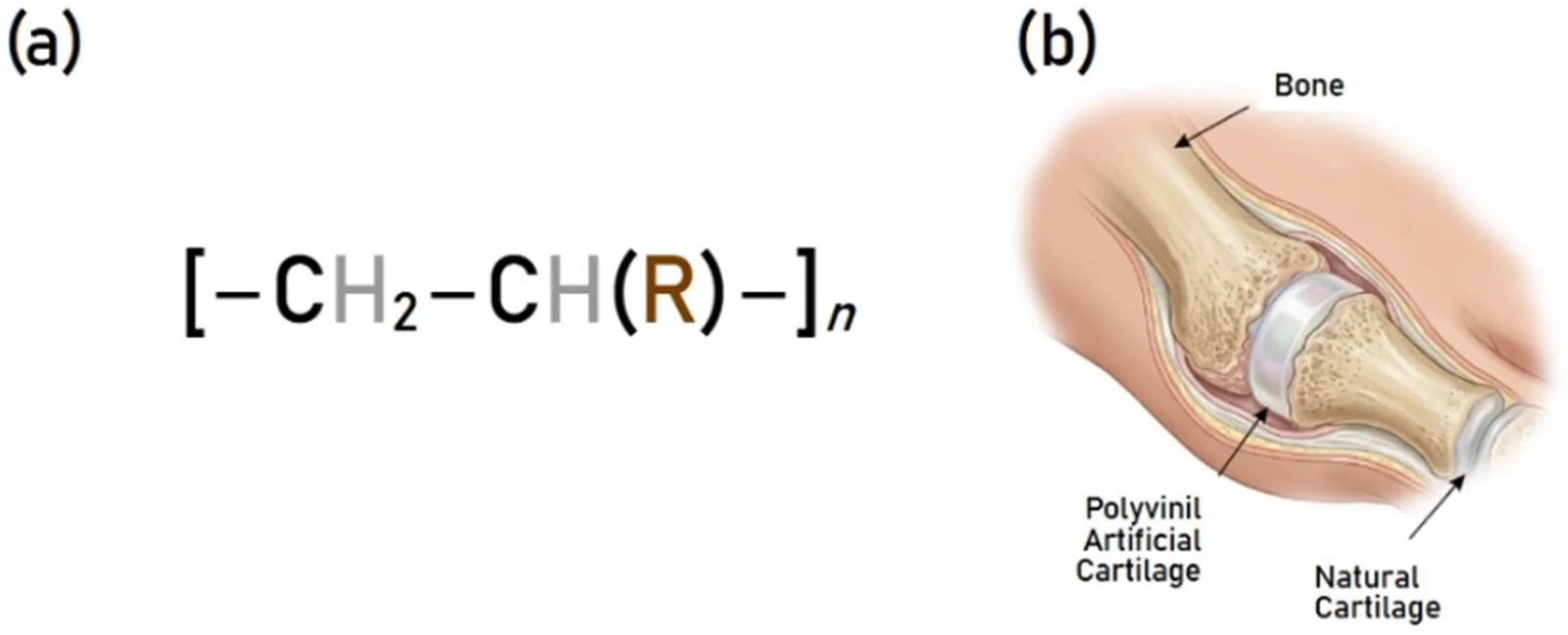
(**a**) Generic repeating structural unit of polyvinyl polymers, characterized by a vinyl backbone with variable pendant functional groups. (**b**) Example of a biomedical application: poly(vinyl alcohol) (PVA) hydrogel used as an artificial cartilage substitute.

Polyvinyl polymers consist of a saturated carbon–carbon backbone derived from vinyl monomers, with functional side groups that largely determine their physicochemical behavior. The nature of the pendant substituent strongly influences degradation susceptibility. For example, PVC contains chlorine substituents that can undergo dehydrochlorination under thermal, mechanical, or oxidative stress, leading to the formation of conjugated double bonds along the backbone [251]. This process not only causes discoloration but also destabilizes the polymer structure. As shown in Figure 9, polyvinyl polymers share a saturated carbon–carbon backbone with variable pendant functional groups (Figure 9a), which strongly influence their physicochemical properties and degradation behavior. Figure 9b shows a representative application of PVA hydrogel as an artificial cartilage substitute, where high water content, flexibility, and tissue-like mechanical behavior are desirable.

Oxidative degradation represents the principal chemical vulnerability in most polyvinyl systems. Reactive oxygen species may induce chain scission or trigger substituent elimination reactions, progressively reducing molecular weight and mechanical integrity [252]. In flexible medical-grade PVC, degradation is often compounded by plasticizer migration or leaching, which increases stiffness and reduces ductility over time [253].

Within the degradation network framework, polyvinyls behave as oxidation-initiated systems with strong mechanical amplification. Chemical destabilization of the backbone increases brittleness and lowers resistance to crack initiation. Mechanical loading generates microcracks that enhance oxygen diffusion into the bulk, reinforcing oxidative attack in a positive feedback loop. In plasticized systems, the progressive loss of plasticizer further increases modulus, exacerbating stress concentration and fatigue susceptibility.

Clinically, degradation may manifest as embrittlement, discoloration, reduced flexibility, and eventual cracking under cyclic mechanical stress. Design strategies to interrupt these pathways include stabilizer incorporation (heat and light stabilizers), optimized plasticizer selection [253], and surface barrier treatments to reduce oxidative exposure.

*Typical biomedical applications:*

- Blood bags and IV tubing (PVC) [254]
- Catheters and flexible tubing [255]
- Drug delivery films [256]
- Wound dressings (PVA-based) [257]
- Encapsulation materials [258]

*Chemistry:* Carbon–carbon backbone with pendant functional groups (–Cl, –OH, –OAc)

*Intrinsic vulnerability:* Oxidation (Ox) dominant; substituent instability (e.g., dehydrochlorination in PVC) [259]

*Network role:* Oxidation-initiated system with chemically driven backbone destabilization

*Failure phenotype:*

- Embrittlement [259]
- Plasticizer leaching [259]
- Fatigue-assisted cracking [260]

### 6.8. Polyimides

Polyimides are high-performance polymers characterized by rigid aromatic backbones incorporating cyclic imide groups. Their conjugated, thermally stable structure provides exceptional mechanical strength, chemical resistance, and dimensional stability. These properties make them particularly attractive for long-term implantable electronics, neural probes, and microfabricated biomedical devices where thin-film integrity and dielectric performance are critical [261].

The aromatic backbone and imide ring confer substantial resistance to hydrolysis and oxidative degradation under physiological conditions. However, while chemically stable in bulk form, polyimides can exhibit vulnerability at interfaces and under sustained mechanical stress. In thin-film configurations—common in bioelectronic applications—cyclic mechanical loading, bending, and micromotion relative to surrounding tissue may induce microcrack formation or interfacial delamination [262].

**Figure 10:**
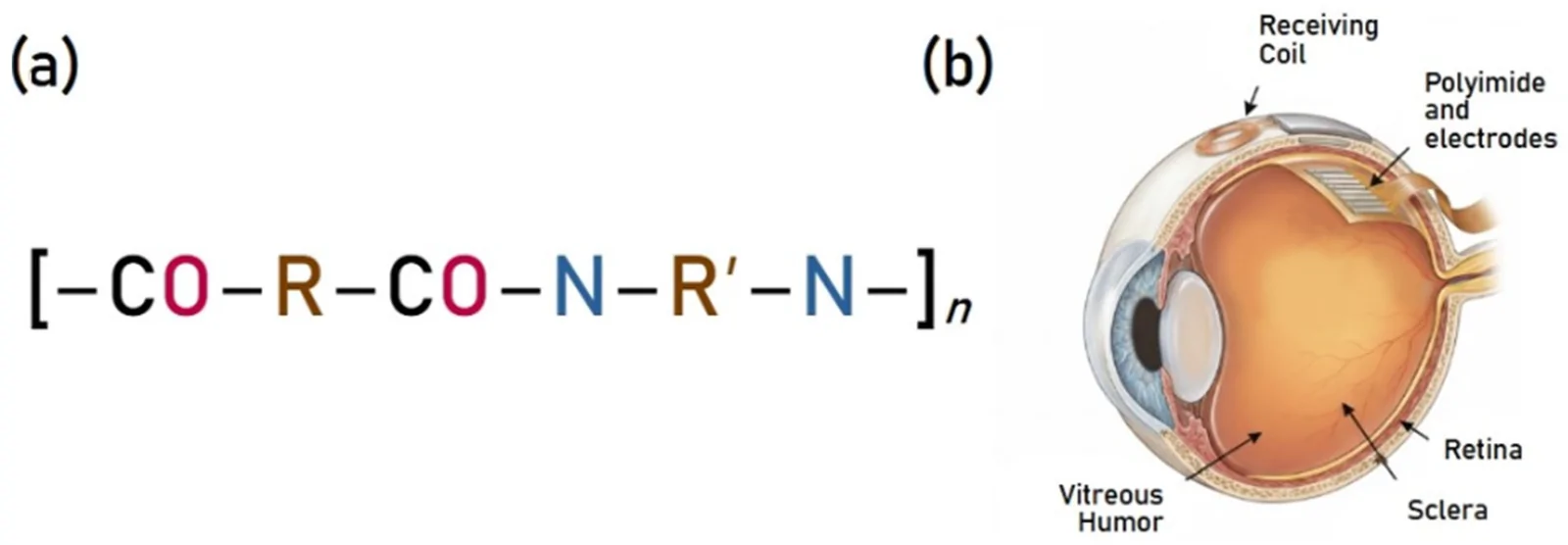
(**a**) Generic repeating structural unit of polyimides, characterized by aromatic backbones containing imide linkages. (**b**) Example of a biomedical application: polyimide substrates used for microelectrode arrays in ocular implants.

Figure 10 highlights the characteristic structure and biomedical relevance of polyimides. The rigid backbone containing imide functionalities (Figure 10a) contributes to their high chemical resistance, dimensional stability, and dielectric performance. Figure 10b shows a representative application of polyimide substrates in ocular microelectrode arrays, where thin-film flexibility, electrical insulation, and long-term structural stability are critical.

Within the degradation network framework, polyimides are best described as mechanically dominated systems with limited chemical amplification. Mechanical fatigue acts as the primary initiating mechanism, particularly in flexible devices subjected to repeated deformation. Microcracks may permit localized fluid ingress, potentially enabling slow hydrolytic attack at exposed imide groups, especially at elevated temperatures or over extended implantation periods. However, such chemical processes are typically secondary to and spatially confined relative to the dominant mechanical damage pathways.

Clinically, degradation manifests primarily as fatigue crack propagation, delamination from metallic conductors or substrates, and gradual compromise of dielectric properties rather than bulk chemical erosion. Strategies to mitigate degradation focus on improving interfacial adhesion, optimizing film thickness and flexibility, reducing stress concentrations, and incorporating barrier layers to limit moisture penetration.

*Typical biomedical applications:*

- Neural interfaces and electrode insulation [263]
- Flexible implantable electronics [264]
- Microfabricated biosensors [265]

*Chemistry:* Aromatic backbone with cyclic imide groups (–CO–NH–CO–) integrated into rigid heteroaromatic structures

*Intrinsic vulnerability:* Mechanical fatigue (Me) dominant; hydrolysis (Hy) limited but possible under prolonged exposure [266]

*Network role:* Mechanically dominated system with high chemical resistance

*Failure phenotype:*

- Fatigue crack propagation [267]
- Interfacial delamination [262]
- Gradual loss of dielectric integrity [268]

### 6.9. Natural Polymers

Natural polymers are composed of biological macromolecules such as proteins (e.g., collagen, silk fibroin) or polysaccharides (e.g., chitosan, alginate, hyaluronic acid). Their backbones contain peptide or glycosidic bonds that are specifically recognized and cleaved by endogenous enzymes. This intrinsic bio-recognition confers excellent biocompatibility and bioactivity but also renders these materials highly susceptible to enzymatic degradation in vivo [269].

Unlike most synthetic polymers, degradation of natural polymers is often regulated by cellular activity and local biological conditions. Enzymes such as collagenases, lysozyme, or matrix metalloproteinases can selectively cleave backbone bonds, leading to either surface erosion or bulk degradation depending on crosslink density and material architecture. Hydrolytic processes may contribute, particularly in hydrated environments, but typically play a secondary role to enzymatic attack [270].

Within the degradation network framework, natural polymers represent enzymatic hub systems. Protein adsorption at material surfaces can facilitate enzyme localization and retention, amplifying degradation rates [137]. Enzymatic cleavage reduces structural integrity and mechanical strength, increasing susceptibility to deformation and microdamage under physiological loading. Mechanical damage, in turn, enhances enzyme penetration and substrate accessibility, reinforcing a biologically driven amplification loop. Swelling phenomena may further modulate mechanical stability, especially in hydrogel systems.

Clinically, this degradation phenotype manifests as controlled bioresorption in regenerative applications or, if insufficiently stabilized, premature loss of mechanical integrity. Design strategies therefore focus on tuning crosslink density, chemical modification of reactive groups, blending with synthetic polymers, or incorporating enzyme inhibitors to modulate degradation kinetics and interrupt excessive amplification pathways.

*Main examples:*

- Collagen
- Gelatin
- Chitosan
- Alginate
- Hyaluronic acid
- Silk fibroin

*Typical biomedical applications:*

- Tissue engineering scaffolds [271]
- Wound dressings [272]
- Drug delivery matrices [273]
- Injectable hydrogels [274]

*Chemistry:* Polypeptide or polysaccharide backbones with enzymatically cleavable glycosidic or peptide bonds

*Intrinsic vulnerability:* Enzymatic degradation (En) dominant; hydrolysis (Hy) secondary [145]

*Network role:* Enzymatic hub system with strong bio-environmental coupling

*Failure phenotype:*

- Rapid bioresorption [145]
- Loss of mechanical integrity [275]
- Swelling-induced deformation [276]

### 6.10. Adaptive, Biohybrid, and Living Polymeric Biomaterials

The conventional polymer classes discussed above are often treated as passive materials whose functionality decreases monotonically as chemical and mechanical damage accumulates. Emerging biomaterials challenge this assumption. Self-healing polymers, dynamic covalent networks, biohybrid constructs, and engineered living materials can reorganize, repair, or actively remodel in response to their environment [275,277].

Self-healing polymeric biomaterials restore at least part of their network connectivity after damage. Healing may be mediated by reversible hydrogen bonding, host-guest interactions, metal-ligand coordination, hydrophobic association, ionic interactions, or dynamic covalent reactions [278,279,280]. Rapidly self-healing hydrogels and injectable shear-thinning networks have shown that reversible physical interactions can permit flow through a needle followed by recovery of solid-like behavior [278,279,281]. In an implant, this capacity could blunt crack-tip stress, close microdefects, preserve barrier function, or reduce the rate at which mechanical damage exposes fresh chemically reactive surfaces [275,279]. However, healing demonstrated after a single macroscopic cut is not equivalent to fatigue resistance. Clinically relevant evaluation should quantify recovery after repeated damage in hydrated, protein-containing, and oxidative environments and should distinguish geometric closure from restoration of strength, toughness, permeability, and interfacial adhesion [275,282].

Dynamic covalent polymers provide a particularly versatile route to reactivity because their crosslinks can exchange while remaining covalently connected. Some polymer chemistries can generate injectable, self-healing, stress-relaxing, and reprocessable networks [283,284,285]. Hydrogels combining two dynamic covalent chemistries have enabled triggered gelation, ligand incorporation, and cytocompatibility [284], while systematic variation in dynamic crosslink parameters can tune injectability and recovery [285]. Fully synthetic dynamic-covalent networks can also be designed to exhibit biomimetic strain-stiffening [286]. These benefits arise from bond exchange, but the same exchange creates a design trade-off: a network that relaxes too rapidly may creep, lose dimensional stability, or permit cargo escape, whereas a network that exchanges too slowly may not heal on the time scale of physiological damage [283,285,287]. The relevant variable is therefore not simply crosslink density, but the distribution of bond lifetimes relative to loading, diffusion, inflammation, and tissue-remodeling time scales.

Biohybrid materials combine a synthetic or processed polymer phase with biologically derived structural or functional components. Examples include synthetic networks reinforced or functionalized with decellularized extracellular matrix [288,289]. Patient-specific extracellular-matrix scaffolds demonstrate how clinical imaging, biopolymer processing, and controlled microarchitecture can be integrated into conformal constructs [290]. Biohybrid muscle systems and decellularized-matrix composites further show that polymer mechanics, cell organization, perfusion, and matrix-derived signaling can be designed together [291,292].

Engineered living materials move this concept further by embedding viable, often genetically programmed, cells within or as the material [293,294]. Bacteria have been engineered to secrete or display self-interacting proteins and assemble centimeter-scale matrices whose composition, mechanics, catalytic activity, and regenerative capacity are genetically controlled [294]. Light-based printing of programmed bacteria has enabled spatially organized living materials [295], and tunable hydrogel viscoelasticity has been used to regulate bacterial behavior within confined matrices [296]. Living probiotic hydrogels have also been investigated for infected-wound treatment, where the embedded organisms provide sustained biological activity that a passive drug-loaded polymer cannot reproduce [297,298]. These systems are genuinely non-equilibrium: nutrients, oxygen, metabolites, cell growth, genetic state, matrix mechanics, and host immunity continuously feed back on one another [293,296].

These emerging systems also expand the definition of failure. For dynamic networks, failure may involve bond-exchange fatigue, catalyst or reactive-group depletion, uncontrolled creep, or the release of exchange products [282,283,285]. For biohybrids, variability in biological source material, decellularization, residual immunogenicity, and matrix remodeling become central [288,289,299]. For living materials, viability, genetic stability, biocontainment, horizontal gene transfer, metabolite release, infection risk, sterilization incompatibility, shelf life, and the ability to terminate activity must be controlled [293,300,301]. Regulatory classification may be product-specific and potentially borderline because a system whose principal action depends on viable organisms or cells is not equivalent to a conventional inert medical device [293,300]. These challenges do not diminish the importance of the field; rather, they show that “alive” must be treated as a measurable and governable design attribute.

Self-healing, dynamic covalent, biohybrid, and living biomaterials therefore represent more than additional polymer categories. They change the governing paradigm from selecting a stable material to programming an evolving system [275,277,293]. Their future clinical value will depend on demonstrating that adaptation is reproducible, bounded, and functionally beneficial under realistic biological and mechanical conditions [275,293,300]. When those criteria are met, polymeric biomaterials may no longer be designed merely to resist the body or disappear within it, but to sense, repair, cooperate, and remodel with living tissue [275,293].

## 7. Degradation Susceptibility for the Most Common Biomedical Polymers

Table 3 summarizes, in a compact form, the *qualitative degradation susceptibility* of the most common biomedical polymers across the seven mechanisms considered in this review: mechanical fatigue (Me), wear (We), lipid absorption (Li), oxidation (Ox), protein adsorption (Pr), hydrolysis (Hy), and enzymatic degradation (En). Each entry is a relative susceptibility score on a 1–5 scale (1 = negligible, 5 = high), and asterisks (*) indicate the dominant degradation driver(s) for that polymer under typical physiological conditions.

The values reported in Table 3 should be interpreted as qualitative susceptibility rankings, not as absolute or quantitative degradation rates. Each score reflects the relative tendency of a polymer to undergo a given degradation mechanism compared with other materials in the same table, assuming broadly similar physiological conditions. Because degradation is inherently multiscale and context-dependent, these values do not represent intrinsic constants of the material but rather comparative indicators derived from recurring patterns observed across the literature.

Importantly, the table does not imply that a polymer with a high susceptibility score will always degrade rapidly, nor that a low score guarantees long-term stability. Instead, the rankings highlight which mechanisms are most likely to dominate the degradation profile of each polymer family. For example, polyesters consistently show high susceptibility to hydrolysis, whereas polyolefins are comparatively resistant to hydrolytic cleavage but more sensitive to oxidative pathways. Natural polymers, in contrast, are primarily governed by enzymatic degradation, with hydrolysis or oxidation playing secondary roles.

The table should therefore be read horizontally—examining the characteristic vulnerabilities of each polymer—and vertically, comparing how different materials respond to the same degradation driver. This dual perspective provides a comparative map of degradation archetypes, helping to identify which mechanisms are likely to act as initiating events, which may serve as amplification pathways, and which are largely irrelevant for a given polymer class. Because the scores are qualitative, they are best used to guide mechanistic reasoning, experimental design, and material selection rather than to predict absolute lifetimes or kinetics.

## 8. Design Implications of the Degradation Network Framework

The practical value of a degradation-network framework lies in its ability to explain why material optimization rarely eliminates failure pathways, but more often redistributes vulnerability across competing mechanisms. Crosslinking, antioxidant stabilization, copolymerization, reinforcement, and surface modification should therefore be interpreted as strategies that reshape the degradation network rather than remove it. Their success depends on whether the dominant pathway is shifted toward a mechanism that is slower, more predictable, less biologically disruptive, or better aligned with the intended functional lifetime of the device [79,147,148,319,320,321,322,323].

This design perspective follows directly from the interaction matrix described above: suppressing one active node can decrease a particular degradation route, but it can also expose alternative pathways whose clinical relevance depends on polymer chemistry, processing history, surface state, geometry, loading, and the local biological environment [14,24,147,148,319,320,321,322,323].

### 8.1. Shifting Rather than Eliminating Degradation Pathways

The history of polymeric bearing materials in joint arthroplasty illustrates how the optimization of one degradation pathway can expose or amplify another. Early use of poly(tetrafluoroethylene) (PTFE) was motivated by its low coefficient of friction and chemical inertness [324]. However, these properties did not translate into durable performance under cyclic articulation. PTFE generated large amounts of wear debris, which accumulated in peri-implant tissues and contributed to inflammation, osteolysis, implant loosening, and clinical failure [21,39,40,41,324,325]. In network terms, PTFE demonstrates that chemical inertness is insufficient when the dominant active pathway is mechanically driven wear.

The subsequent adoption of ultra-high-molecular weight polyethylene (UHMWPE) represented a major improvement because of its superior toughness, lower wear rate, and more favorable tribological behavior [36,37,66,68,130,319,320]. Nevertheless, UHMWPE did not eliminate degradation. Instead, its long-term performance became governed by the balance between wear, fatigue, and oxidation. Reinforcement strategies, including fiber incorporation, could improve selected bulk mechanical properties, but they also introduced interfaces and stress-concentration sites that promoted debonding, microcracking, and debris formation [130,148,319].

Gamma irradiation provided a more effective route by inducing crosslinking within UHMWPE. Highly crosslinked polyethylene substantially reduced wear debris generation in articulating surfaces [34,37,319,320,321]. However, irradiation also generated residual free radicals, particularly within crystalline regions [21,31,38,321]. These radicals increased susceptibility to long-term oxidative degradation, leading to chain scission, embrittlement, and reduced fracture resistance during in vivo service [21,31,35,38,79,321,322,323].

Thermal remelting and annealing were introduced to reduce residual free radicals and improve oxidative stability [31,34,38,321,322,323]. These approaches improved the balance between wear resistance and oxidation resistance, although they also required trade-offs with crystallinity and mechanical strength [31,34,38,321,322,323]. Vitamin E stabilization further refined this balance by acting as a radical scavenger while preserving more of the polymer microstructure than full remelting [321,322,323,326]. However, vitamin E incorporation may affect crosslink density, antioxidant distribution, and long-term local protection, indicating that even successful stabilization strategies introduce new variables into the degradation network [321,322,323,326].

### 8.2. Bulk Modification Strategies

Bulk modification strategies act by changing the internal architecture of the polymer, including chain mobility, crystallinity, molecular weight distribution, crosslink density, radical concentration, and phase morphology. These changes can suppress a dominant degradation pathway, but they also modify the susceptibility of the material to alternative chemical or mechanical processes [163,164,184,187,188,189,319,320,321,322,323].

Crosslinking is a representative example of this trade-off. Increasing network density can reduce chain mobility, wear, dissolution, or swelling, depending on the polymer system. At the same time, excessive crosslinking may reduce ductility, alter fatigue behavior, or make oxidative and fracture-related processes more consequential under long-term service conditions [34,35,36,37,79,319,320,321,322,323]. In the context of UHMWPE, this trade-off is particularly evident because increased crosslink density improves wear resistance while processing history and residual radicals influence oxidation susceptibility [21,31,38,319,320,321,322,323].

Reinforcement strategies follow a similar logic. Fibers, fillers, or porous reinforcements can improve stiffness, strength, or toughness, but they also introduce interfaces where debonding, stress concentration, water ingress, or microcrack initiation may occur [130,148,171,319]. Reinforcement should therefore be evaluated not only by the mechanical property it improves, but also by the new interfacial or fatigue-sensitive pathways it may introduce into the degradation network.

Copolymerization provides a broader route for redistributing degradation susceptibility because it can tune crystallinity, hydrophilicity, chain mobility, oxidative stability, mechanical toughness, and hydrolysis rate within the same macromolecular architecture [163,164,184,187,188,189]. In biodegradable systems, poly(lactic-co-glycolic acid) (PLGA) illustrates this principle: the relative proportion of lactic and glycolic units controls crystallinity, water uptake, and the rate of hydrolysis, allowing degradation kinetics to be adjusted while maintaining broadly similar chemical functionality [105,142,164,303].

From a network perspective, copolymerization, crosslinking, stabilization, and reinforcement do not remove nodes from the degradation map. Rather, they alter the probability that specific hubs become dominant under physiological conditions [14,147,148,160,163]. The design objective is therefore not a universally stable polymer, but a material whose vulnerabilities remain slow, predictable, or functionally acceptable in the intended application.

### 8.3. Application-Specific Success and Failure

A central implication of the network perspective is that polymer performance cannot be judged independently of the application environment. The same material may be clinically successful when its dominant degradation pathways remain slow or functionally irrelevant, but may fail when the device geometry, loading regime, or biological milieu activates a critical pathway. Material selection should therefore be based not only on intrinsic chemical stability, but on the expected hierarchy of degradation mechanisms under the intended service conditions.

PTFE provides a clear example. Early attempts to employ PTFE as a bearing material in joint arthroplasty resulted in severe failure due to extensive wear debris generation under cyclic articulation [324,325]. The particulate material produced during articulation triggered inflammatory responses and osteolysis, ultimately leading to implant loosening [20,39,40,324,325]. However, PTFE remains successful in other biomedical applications where such tribological stresses are absent. Expanded PTFE is widely used in vascular grafts and surgical membranes, where chemical inertness, low surface energy, and resistance to biological attack provide long-term stability [327,328,329]. In these applications, the degradation mechanism responsible for failure in joint prostheses does not become dominant.

A similar application-dependent behavior can be observed in poly(ether ether ketone) (PEEK). PEEK exhibits high mechanical strength, chemical stability, and radiolucency, which support its use in spinal cages and other load-bearing orthopedic implants [71,126,330]. Nevertheless, when considered as an articulating surface in joint replacements, PEEK has shown less favorable tribological behavior, including higher wear rates than established bearing materials [331,332,333]. The polymer is therefore not intrinsically unsuitable; its vulnerability becomes critical when the application activates a mechanically dominated degradation pathway.

Poly(methyl methacrylate) (PMMA) illustrates the same principle. PMMA has been used successfully for decades as a bone cement in orthopedic surgery, where it acts as a space-filling and load-distributing material rather than as a structural implant [244,334,335]. In this role, brittleness and limited fatigue resistance can be acceptable because the cement primarily anchors metallic components [244,334,335]. However, PMMA is less suitable as a load-bearing structural material in joint replacements, where cyclic mechanical stresses can induce cracking and fatigue failure [74,242,334].

Polyethylene terephthalate (PET) provides an additional example. PET fibers have been widely used in vascular grafts and surgical meshes because of their tensile strength and chemical stability in physiological environments [336,337,338]. However, when PET was explored as a ligament replacement material, repeated bending and frictional loading contributed to progressive mechanical degradation and wear, ultimately limiting several early designs [336,337,338].

Even UHMWPE illustrates this application-dependence. In joint arthroplasty, it performs well as a bearing surface when paired with appropriate counterfaces and processing methods such as crosslinking [34,36,37,66,68,319,321,322]. The same polymer would be less appropriate in environments where high oxidative activity becomes the dominant boundary condition, because oxidation can shift the failure mode toward embrittlement and fatigue-assisted fracture [34,36,37,66,68,319,321,322].

Together, these cases show that long-term stability rarely derives from intrinsic material perfection. It arises from alignment between material susceptibility and the stresses imposed by the device environment. By shifting the hierarchy of degradation mechanisms through strengthening, copolymerization, stabilization, or surface engineering, material design can extend functional lifetime even though the underlying degradation network remains active [14,79,128,172,186,319,320,321,339,340,341,342,343,344].

### 8.4. Surface Engineering as Interfacial Network Control

Surface engineering can be interpreted as a strategy for controlling the boundary layer of the degradation network. Because protein adsorption, cell adhesion, oxidative attack, hydrolysis, lubrication, and wear often initiate at the implant-environment interface, surface modification can alter the early conditions that determine which downstream pathways become dominant [23,24,172,186,345,346,347,348].

This role is distinct from bulk strengthening. Surface modification typically affects only the outermost nanometers or micrometers of a device and therefore rarely provides major improvements in bulk strength or structural fatigue resistance [24,28,345,346,347]. Nevertheless, it can strongly influence biological performance by modulating protein adsorption, cell adhesion, inflammatory activation, bacterial colonization, surface energy, and interfacial transport [5,22,23,24,28,84,90,180].

The value of surface engineering is evident in PEEK. Although PEEK has favorable mechanical properties, chemical stability, and radiolucency, its bioinert surface can limit direct bone integration in orthopedic applications [71,126,330,345,346,347,348]. Plasma treatments and surface functionalization have therefore been used to increase surface energy and introduce reactive groups that promote osteoblast adhesion and proliferation while preserving bulk properties [173,345,346,347,348].

A related example involves hydrophobic polymers such as PTFE. Plasma-based activation, chemical grafting, and hydrophilic coatings can introduce functional groups that improve protein adsorption and cellular attachment while preserving the chemical stability of the bulk material [174,179,186,327,328,349,350,351]. In these cases, the goal is not to change the intrinsic polymer backbone, but to redirect the interfacial biological response.

Surface-grafted UHMWPE systems further illustrate both the potential and the limitations of this approach. The Aqualà^®^ polyethylene system was developed to reduce friction and suppress oxidative degradation through surface grafting [339,340,341,342,343,344]. Initial reports suggested improved tribological behavior, but subsequent investigations indicated that the grafted layer could degrade or detach under physiological loading, exposing the underlying polyethylene substrate and, in some cases, increasing oxidative susceptibility relative to conventional materials [339,340,341,342,343,344].

Thus, surface modification should be evaluated as interfacial network control rather than as a permanent solution to degradation. Its effectiveness depends on the stability of the modified layer, the mechanical and chemical conditions at the interface, and whether exposure of the underlying polymer would reactivate a more damaging pathway [23,24,81,186,343,344,349].

### 8.5. Regulatory Translation, Implant Design, and Personalized Polymeric Biomaterials

The future of polymeric biomaterials will depend not only on discovering new chemistries, but also on translating dynamic material behavior into verifiable device performance. For an implant, degradation changes the identity of the material over time: molecular weight, crystallinity, crosslink density, surface chemistry, additive concentration, debris generation, and mechanical integrity may all evolve simultaneously [82,83]. ISO 10993 provides complementary frameworks for chemical characterization and toxicological risk assessment of device constituents, manufacturing residues, extractables, leachables, and degradation products [111]. ISO 10993 is also useful for identifying degradation products from finished polymeric devices, but its stated scope excludes several coupled phenomena central to long-term implant performance, including mechanical stress, wear, ionizing radiation, enzymes, proteins, and cellular activity [111]. Consequently, compliance with a minimum standard battery should not be interpreted as proof that a polymer–device system has been challenged under all clinically relevant degradation networks. A risk-based testing strategy should begin with the intended clinical function and the time window over which that function must be retained [111]. The European Medical Device Regulation requires consideration of compatibility with tissues and body fluids, the influence of manufacturing processes, and material properties such as strength, wear resistance, fatigue strength, and surface characteristics. It also requires control of risks associated with particles, wear debris, and degradation products [111,352,353]. These requirements align closely with the functionality-retention framework developed in this review. For a bioresorbable fixation device, the relevant question is not whether mass is eventually lost, but whether mechanical support remains above the clinical threshold until tissue healing has transferred the load. For a permanent elastomeric lead or catheter, the design target is a bounded change in compliance, fatigue resistance, surface integrity, and extractable profile over the intended service life [14,82,83]. In both cases, accelerated aging should be mechanistically justified and linked to real-time or in vivo evidence; excessive temperature, oxidant concentration, pH, or mechanical amplitude can change the dominant pathway and produce an apparently conservative test that is not mechanistically representative [14,354].

Implant design is therefore an intervention in the degradation network. Geometry determines stress concentration, diffusion length, surface-to-volume ratio, and the probability of local autocatalysis. Porosity and architecture control load transfer, fluid transport, cellular infiltration, and the accessibility of enzymes or reactive oxygen species. Processing changes crystallinity, residual stress, molecular orientation, interfacial defects, and additive distribution, while sterilization may generate radicals, chain scission, crosslinking, or altered surface chemistry. Surface treatments can reduce fouling or inflammation but can also introduce delamination, transport barriers, or new chemical species. Rational design should optimize these variables as a coupled system and define an application-specific design space within which functionality retention, degradation products, and biological response remain acceptable [14,82,83]. Reviews of biodegradable implant translation similarly emphasize that host response, degradation mechanism, device architecture, and manufacturing history must be considered together rather than sequentially [83].

Patient-specific and point-of-care manufacturing makes this coupling more explicit. Imaging-derived implants have been demonstrated for pediatric airway support, cranio-maxillofacial reconstruction, and patient-specific regenerative scaffolds [355,356,357]. These studies show that personalization can improve anatomical conformity and enable control of local wall thickness, porosity, channel geometry, fiber orientation, and scaffold distribution. At the same time, every geometry becomes a new mechanical and transport problem [356,357]. Translation therefore requires a validated design envelope that links image acquisition and segmentation, software operations, allowable geometric features, material lot, printing parameters, post-processing, cleaning, packaging, sterilization, and release testing.

Personalization should be understood at three interconnected levels. Anatomical personalization adapts macroscopic shape to the defect and can reduce dead space, micromotion, or nonuniform load transfer. Mechanical personalization adapts stiffness, anisotropy, compliance, degradation time, and local architecture to the tissue and expected loading history. Biological personalization aims to match the patient-specific host environment, including inflammatory state, age, comorbidities, immune phenotype, metabolic status, healing capacity, and local microbiology. Patient-specific extracellular-matrix scaffolds and composite regenerative systems illustrate how shape, microarchitecture, and biological composition can be tailored together [290,358,359]. However, biological personalization is more difficult to validate than geometric personalization because the host response is probabilistic and time-dependent. Recent work showing that local immunometabolic cues reorganize the cell populations surrounding implanted polylactide reinforces the need to treat patient biology as a design variable rather than as uncontrolled background noise [360].

Greater personalization also increases regulatory and quality-system burdens. Under the EU MDR, a custom-made device is specifically manufactured according to a written prescription that defines patient-specific design characteristics for the exclusive use of an individual patient. In contrast, devices manufactured through standardized or industrial processes and subsequently adapted to individual requirements are not necessarily classified as custom-made devices [352,353]. Post-market surveillance is especially important because rare failures may arise only from specific combinations of anatomy, local biology, loading, and material history [356,357].

However, if degradation mechanisms are coupled, then testing them in isolation can identify intrinsic vulnerabilities but may fail to reproduce the pathway hierarchy that emerges in vivo. More informative protocols should combine mechanism-specific assays with sequential or simultaneous exposure to biologically relevant stressors, including aqueous media, enzymes, reactive oxygen species, protein conditioning, lipid exposure, cyclic loading, and wear [14,82,87,104,105,147,148,354].

For hydrolysis-dominated polyesters, pH-controlled hydrolytic aging should be combined with mechanical testing or cyclic loading when the intended application involves structural support. This is necessary because hydrolysis-driven molecular weight loss can reduce fatigue resistance, while mechanical damage can increase fluid ingress and accelerate further hydrolytic attack [16,76,104,121,140,160,361].

For polyolefin bearing materials, wear tests should be interpreted together with oxidative aging and, where appropriate, biological assessment of wear debris. In joint arthroplasty, mechanically generated particles can activate macrophage responses and reactive oxygen species production, which may reinforce oxidative degradation and further weaken the polymer matrix [20,31,36,37,38,39,40,41,54,79,362].

For polyurethanes and natural polymers, protein conditioning and enzyme exposure should be treated as coupled interfacial variables rather than optional additions. Protein adsorption can localize enzymes, change cell adhesion, and alter inflammatory activation, while enzymatic cleavage can reduce mechanical integrity and increase substrate accessibility [5,90,91,137,145,161,220,269,270,275,276].

For hydrogels and water-sensitive polymers, swelling, plasticization, creep, and fatigue should be evaluated together. Water uptake can modify chain mobility and mechanical response even when backbone scission is limited, meaning that functional failure may occur before substantial chemical degradation or mass loss is observed [184,208,241,311,312].

These combined protocols should not replace standardized biological evaluation and material testing, but they can complement ISO and ASTM frameworks by explicitly reporting the gap between simplified test media and physiological boundary conditions [82,87,111,112,113]. At a minimum, studies intended to support degradation network modeling should report polymer chemistry, processing history, surface treatment, device geometry, environmental composition, mechanical loading conditions, and time-resolved chemical, mechanical, and biological endpoints.

## 9. Artificial Intelligence and Data-Driven Modeling of Polymer Degradation Networks

One of the major limitations of the degradation-network framework proposed in this work lies in the inability to quantitatively determine the interaction coefficients governing the coupling between degradation mechanisms [14,82,363,364,365]. While the interaction matrix provides a qualitative representation of directional amplification effects, the actual magnitude of these interactions remains largely unknown and is expected to depend on polymer chemistry, processing history, geometry, mechanical loading, and local physiological conditions [14,82,363,364,365,366,367].

In principle, however, the growing availability of experimental data in the biomaterials literature offers the possibility of approaching this problem through data-driven methodologies [364,366,368,369,370,371,372,373]. Advances in artificial intelligence (AI), particularly machine learning (ML) and deep learning approaches, may provide powerful tools for identifying hidden relationships between degradation pathways and estimating interaction coefficients from large heterogeneous datasets [364,366,368,370,371,372,374,375].

Unlike traditional mechanistic modeling, which requires explicit formulation of coupled reaction kinetics and transport phenomena, machine learning systems can identify statistical correlations and non-linear dependencies directly from experimental observations [363,364,365,367,368]. Parameters such as molecular weight evolution, mass loss, crystallinity changes, tensile strength reduction, oxidation indices, wear rates, protein adsorption behavior, inflammatory markers, and enzymatic susceptibility could, in principle, be integrated into predictive degradation models trained on large-scale datasets collected across different polymer classes and biomedical applications [363,366,369,371,372,373,376].

Within this context, the interaction coefficients introduced in the previous sections could potentially evolve from purely conceptual parameters into experimentally inferable quantities [363,364,365,366,367,377,378]. Rather than assigning fixed universal values, AI-based approaches may allow these coefficients to be estimated probabilistically as functions of material composition, processing conditions, implantation environment, and time-dependent biological responses [363,364,365,366,367,377,378].

Importantly, the success of such approaches will strongly depend on the quality, consistency, and granularity of the available experimental data [365,366,369,373,376,379,380,381,382]. At present, degradation studies are often reported using highly simplified testing conditions, incomplete environmental descriptions, or insufficient characterization of coupled mechanical and chemical phenomena [14,82,363,365,366]. In many cases, only isolated degradation endpoints are measured, making it difficult to reconstruct the dynamic interactions between mechanisms [14,82,363,365,366].

For this reason, future studies should aim to provide more comprehensive and standardized datasets, including [14,82,363,365,366,369,371,376,379,382,383,384]:

- Detailed polymer chemistry, copolymer composition, molecular architecture, and processing history;
- Crystallinity, molecular weight distribution, crosslink density, additives, and surface treatments;
- Device geometry, surface area, porosity, and relevant transport length scales;
- Mechanical loading conditions, wear configurations, fatigue protocols, strain rates, and stress amplitudes;
- Environmental parameters, including pH, ionic strength, protein content, lipid content, reactive oxygen species, enzymatic activity, media renewal, and flow;
- Time-resolved chemical, mechanical, structural, interfacial, and biological degradation markers.

The adoption of standardized reporting practices would not only improve reproducibility but also facilitate the development of robust machine learning models capable of identifying degradation archetypes and predicting long-term implant behavior across polymer families [363,364,366,369,372,378,379,384].

Ultimately, artificial intelligence may enable a transition from descriptive degradation studies toward predictive biomaterials science, where the complex interplay between hydrolysis, oxidation, fatigue, wear, enzymatic attack, and biological responses can be modeled as an evolving multidimensional system rather than as isolated mechanisms. In this perspective, the framework proposed in this review may serve as a conceptual foundation for future AI-assisted approaches aimed at quantitatively mapping the dynamic biocompatibility landscape of polymeric biomaterials [363,364,365,366,367,368,372].

## 10. Conclusions

Polymeric biomaterials represent one of the clearest examples of dynamic biocompatibility, where long-term performance is not determined by a single intrinsic material property, but rather by the continuous and evolving interaction between polymer chemistry, mechanics, biology, and the surrounding physiological environment. Unlike traditional views that describe degradation mechanisms as isolated and independent phenomena, the framework proposed in this review interprets polymer degradation as an interconnected and directional network of amplification pathways, in which hydrolysis, oxidation, enzymatic attack, wear, protein adsorption, lipid absorption, and mechanical fatigue continuously influence one another over time.

Within this perspective, degradation should not be understood simply as material loss, but as the progressive evolution of functionality under biologically active conditions. Different polymer classes exhibit characteristic degradation archetypes governed by dominant mechanisms, while strengthening strategies, copolymerization, and surface modifications rarely eliminate degradation altogether. Instead, they usually shift the hierarchy of active pathways. This network-based interpretation helps explain why the same polymer may perform successfully in one biomedical application while failing in another, depending on whether the local mechanical and biochemical environment activates a critical degradation route.

At present, the interaction coefficients and higher-order coupling terms proposed in this work remain largely qualitative. Their quantitative determination is limited by the complexity of physiological environments and by the fragmented nature of available experimental data. Nevertheless, the existing literature contains extensive information on degradation kinetics, environmental conditions, mechanical loading, inflammatory responses, and long-term clinical performance across many polymeric systems. If organized into standardized and interoperable datasets, these data could support semi-quantitative and probabilistic models of polymer degradation.

For the first time, the emergence of machine learning and artificial intelligence methods offers the possibility of transforming this massive but raw body of knowledge into semi-quantitative degradation frameworks. Although the available data are heterogeneous, incomplete, and often generated under non-standardized experimental conditions, they may still contain sufficient statistical structure to estimate approximate interaction magnitudes, identify dominant degradation pathways, and establish realistic orders of magnitude for the coefficients proposed in the present model. In this sense, the goal should not be perfectly deterministic prediction, but the development of adaptive models capable of identifying dominant degradation pathways and estimating their relative importance under biologically relevant conditions.

Importantly, this possibility also highlights the need for a new generation of experimental studies designed not only to characterize materials individually, but also to generate reproducible, standardized, and interoperable datasets suitable for future computational analysis. If future research increasingly adopts rigorous reporting of environmental conditions, degradation metrics, mechanical loading, and temporal evolution, it may eventually become possible to construct predictive degradation maps for polymeric biomaterials with progressively increasing accuracy.

Ultimately, the long-term goal is not simply to observe or slow polymer degradation, but to learn how to rationally control it. Achieving such control would represent a major shift in biomaterials science, enabling polymeric implants whose degradation behavior is intentionally programmed, dynamically adaptive, and functionally synchronized with biological healing and tissue regeneration processes.

## Figures and Tables

**Table 1:** Representative physicochemical conditions in selected anatomical environments relevant to implanted biomaterials, including typical ranges of pH, ionic strength, protein and lipid concentrations, dissolved oxygen levels, and predominant enzymatic species. The table also summarizes the principal degradation drivers expected in each environment, highlighting how local physiological conditions influence the dominant mechanisms governing polymer degradation in vivo.

Anatomical Environment	pH	Ionic Strength (mM)	Protein Concentration (g/L)	Lipid Concentration (mg/dL)	Dissolved O_2_ (mmHg)	Key Enzymes Present [92]	Typical Degradation Drivers
Blood plasma [93]	7.35 – 7.45	150	60–80	500–700	75–100	Esterases [94], oxidases [95]	Oxidation, enzymatic hydrolysis
Interstitial fluid [96]	7.0 – 7.4	120 – 140	10–20	100–300	20–40	Proteases [97], esterases [94]	Hydrolysis, enzymatic degradation
Synovial fluid [98]	7.2 – 7.4	100 – 120	25–30	200–400	30–50	Hyaluronidase [99], lipases [100]	Oxidation, hydrolysis
Subcutaneous tissue fluid [101]	7.0 – 7.3	120 – 140	5–15	100–200	10–30	Esterases [94], proteases [97]	Hydrolysis, mild oxidation
Inflamed tissue/macrophage environment [102]	6.5 – 7.0	Variable	20–40	200–400	<20	Peroxidases [103], hydrolases [100]	Oxidation, enzymatic degradation

**Table 2:** Directed interaction matrix illustrating the qualitative amplification effects among degradation mechanisms in polymeric biomaterials. Each element $I_{ij}$ represents the extent to which mechanism ***i*** (row) exacerbates mechanism ***j*** (column), highlighting the directional nature of degradation interactions. The diagonal elements are not applicable. Interaction strength is classified qualitatively as ○ (negligible interaction), ◗ (weak facilitation), ● (strong synergistic amplification). Abbreviations: Me—mechanical fatigue; We—wear; Li—lipid absorption; Ox—oxidation; Pr—protein adsorption; Hy—hydrolysis; En—enzymatic degradation. Protein adsorption and lipid absorption are included in the matrix not as degradation reactions per se, but as interfacial and transport-mediated modulators that can redirect subsequent degradation pathways by altering enzyme localization, immune-cell adhesion, lubrication, swelling, plasticization, and molecular transport.

	Me	We	Li	Ox	Pr	Hy	En
**Me**	—	●	○	◗	○	◗	○
**We**	◗	—	○	◗	◗	◗	◗
**Li**	◗	○	—	○	○	◗	○
**Ox**	●	◗	○	—	○	◗	○
**Pr**	○	○	○	○	—	◗	●
**Hy**	●	◗	○	◗	○	—	◗
**En**	◗	○	○	○	○	◗	—

**Table 3:** Qualitative vulnerability of common biomedical polymers across seven mechanisms: mechanical fatigue (Me), wear (We), lipid absorption (Li), oxidation (Ox), protein adsorption (Pr), hydrolysis (Hy), and enzymatic degradation (En). Scores (1–5) represent relative, non-quantitative tendencies derived from comparative literature patterns and go from 1, when the phenomenon is negligible to 5 when it is dominant/very high; asterisks (*) indicate the dominant degradation pathway(s) typically observed for each polymer family under physiological conditions. The table is intended as a comparative map of degradation archetypes rather than a predictor of absolute degradation rates.

Polymer Category/Example	Me	We	Li	Ox	Pr	Hy	En	Comments/Notes
**PLA**	**5** *	**4** *	1	2	3	5	2	Brittle; poor fatigue and wear resistance; hydrolytic ester cleavage dominant [302]
**PLGA**	4	3	1	2	3	**5** *	3	Hydrolytic copolymer; moderately brittle; enzyme-sensitive [303]
**PCL**	3	2	3	2	3	**4** *	3	Ductile; good fatigue strength; slow hydrolysis [304]
**PGA**	**5** *	4	1	2	3	**5** *	3	Stiff and brittle; rapid hydrolytic degradation [305]
**PHAs (PHB, PHBV)**	4	3	3	3	3	4	**5** *	Brittle bacterial polyesters; enzyme-sensitive surface erosion [306]
**PE** (**UHMWPE**)	1	1	2	4	2	1	1	Outstanding wear resistance; oxidative aging possible under stress [199]
**PP**	2	2	2	**5** *	2	1	1	Good wear resistance; oxidative chain scission dominates [307]
**PB**	3	3	3	4	2	1	1	Flexible but oxidation-susceptible [307]
**PA**	2	2	2	3	3	**4** *	2	Good fatigue and wear resistance; hydrolysis possible in humid conditions [308]
**PU**	3	3	3	**5** *	3	2	2	Fatigue-tolerant elastomer; oxidation of soft segments dominates [226]
**PDMS (Silicone)**	1	2	**5** *	4	**5** *	1	2	Extremely fatigue-resistant; absorbs lipids and proteins [309]
**PMMA**	**4** *	**4** *	2	3	**4** *	1	1	Brittle; poor fatigue/wear performance; protein fouling [310]
**pHEMA**	3	3	2	2	**3** *	2	2	Soft hydrogel; limited wear but moderate protein adsorption [311]
**PVA**	3	3	2	2	3	**4** *	3	Hydrolysis if not crosslinked; enzymatic attack possible [312]
**PVP**	4	3	1	2	2	1	1	Chemically stable; dissolves physically rather than wearing [313]
**Polyimide (PI)**	1	1	2	4 *	2	1	1	Exceptional wear and fatigue resistance; oxidation limits long-term stability [266]
**Collagen**	5	5	2	2	4	4	**5** *	Rapid enzymatic degradation; hydrolysis secondary [314]
**Gelatin**	5	5	2	2	4	3	**5** *	Easily digested by proteases; loses structure in water [315]
**Chitosan**	4	5	2	2	3	3	**5** *	Degraded by lysozyme and chitosanases [316]
**Alginate**	5	5	1	2	3	2	**4** *	Enzyme-sensitive; otherwise stable in neutral media [317]
**Silk fibroin**	3	2	2	2	3	2	**4** *	High toughness; slow enzymatic degradation [318]

## Data Availability

No new data were created or analyzed in this study. Data sharing is not applicable to this article. During the preparation of this manuscript/study, the authors used DALL·E 3 for the purposes of generation of figures. The authors have reviewed and edited the output and take full responsibility for the content of this publication.

## Abbreviations

The following abbreviations are used in this manuscript:

*AI*	*Artificial Intelligence*
*ASTM*	*American Society for Testing and Materials*
*En*	*Enzymatic degradation*
*FBS*	*Fetal Bovine Serum*
*Hy*	*Hydrolysis/Hydrolytic degradation*
*Li*	*Lipid absorption*
*Me*	*Mechanical fatigue*
*ML*	*Machine learning*
*PBS*	*Phosphate-Buffered Saline*
*PCL*	*Poly(caprolactone)*
*PDO*	*Polydioxanone*
*PDMS*	*Poly(dimethylsiloxane)*
*PE*	*Polyethylene*
*PEEK*	*Poly(ether ether ketone)*
*PGA*	*Poly(glycolic acid)*
*PHA*	*Poly(hydroxyalkanoate)*
*PHB*	*Poly(hydroxybutyrate)*
*PHBV*	*Poly(3-hydroxybutyrate-co-3-hydroxyvalerate)*
*PI*	*Polyimide*
*PLA*	*Poly(lactic acid)*
*PLGA*	*Poly(lactic-co-glycolic acid)*
*PMMA*	*Poly(methyl methacrylate)*
*PP*	*Polypropylene*
*Pr*	*Protein adsorption*
*PTFE*	*Poly(tetrafluoroethylene)*
*PU*	*Polyurethane*
*PVA*	*Poly(vinyl alcohol)*
*PVC*	*Poly(vinyl chloride)*
*PVP*	*Poly(vinylpyrrolidone)*
*ROS*	*Reactive Oxygen Species*
*UHMWPE*	*Ultra-High-Molecular Weight Polyethylene*
*We*	*Wear*
